# Supplementary figures and images for: Environmental redox conditions and strain variation define phenazine-mediated antagonism in co-infecting bacteria
Source: PLoS Biol. 2026 May 20;24(5):e3003809. doi: 10.1371/journal.pbio.3003809 (PMC13197074; doi:10.1371/journal.pbio.3003809)

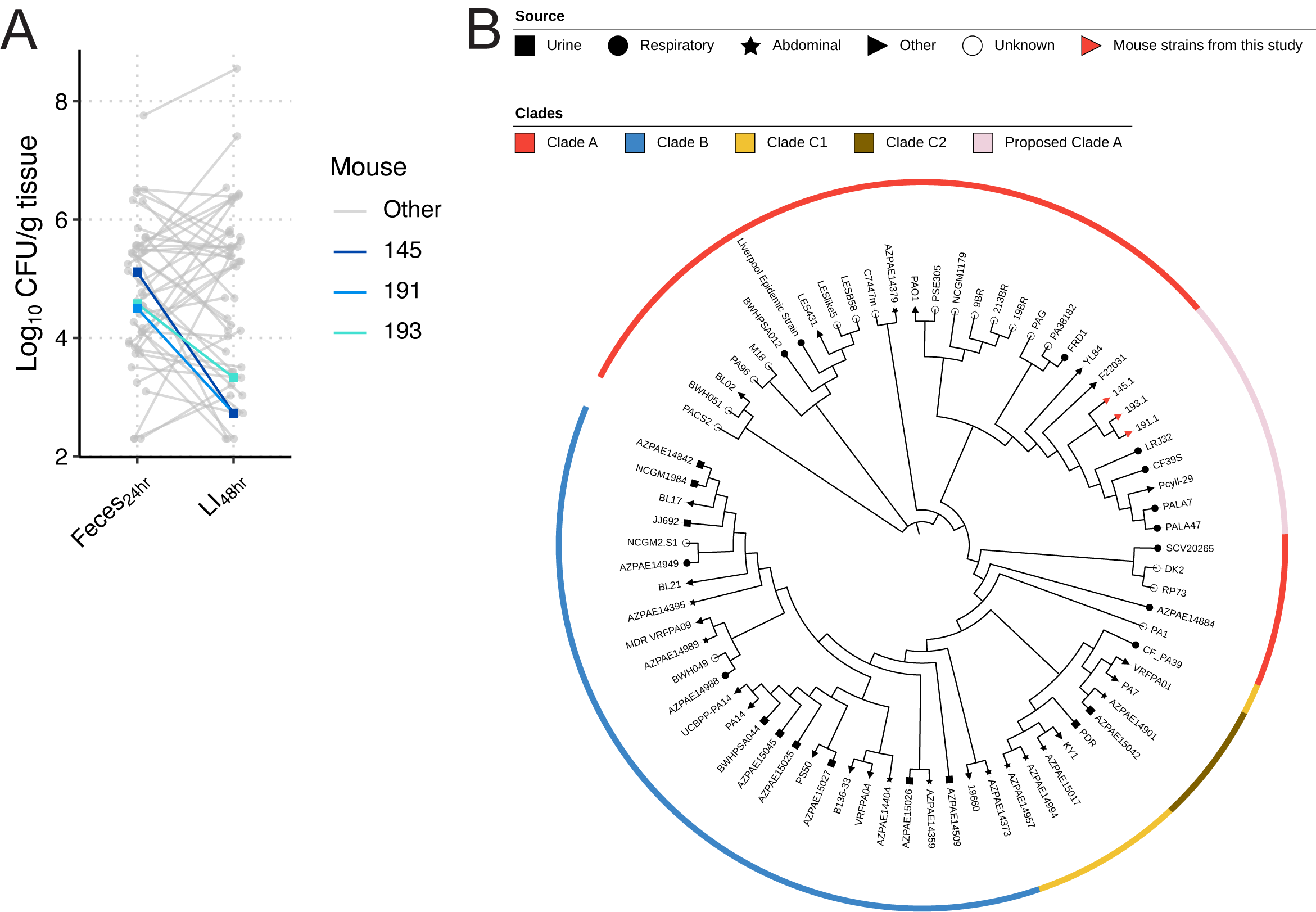

Supplement: S1 Fig — 6- to 8-week-old C57 mice from Taconic Farms were orally inoculated with 108 CFU WT KPPR1 or 13F11. Kp was enumerated from feces at 24 hours and large intestinal contents (LI) 48 hours post-inoculation (A). Pa was isolated from mouse 145, 191, and 193 and subjected to whole genome sequencing. These strains and subset of Pa isolates from a previous study [43] were used to build an approximately-maximum-likelihood phylogenetic tree based on a core genome alignment of these strains to determine if strains 145.1, 191.1, and 193.1 group with Clade A (PAO1 clade), Clade B (PA14 clade), or Clade C (split Pa7 clade). Select Pa strains absent in the previous study that represent ST175 were also included, as strains 145.1, 191.1, and 193.1 were predicted to be most like ST175 using multi-locus sequence typing. The ST175 representative and mouse strains grouped with Clade A, thus they are labeled “Proposed Clade A” (B). The data underlying this Figure can be found in S2 Data and the raw phylogenetic tree data can be found in S4 Data. (TIF) [file pbio.3003809.s001.tif]

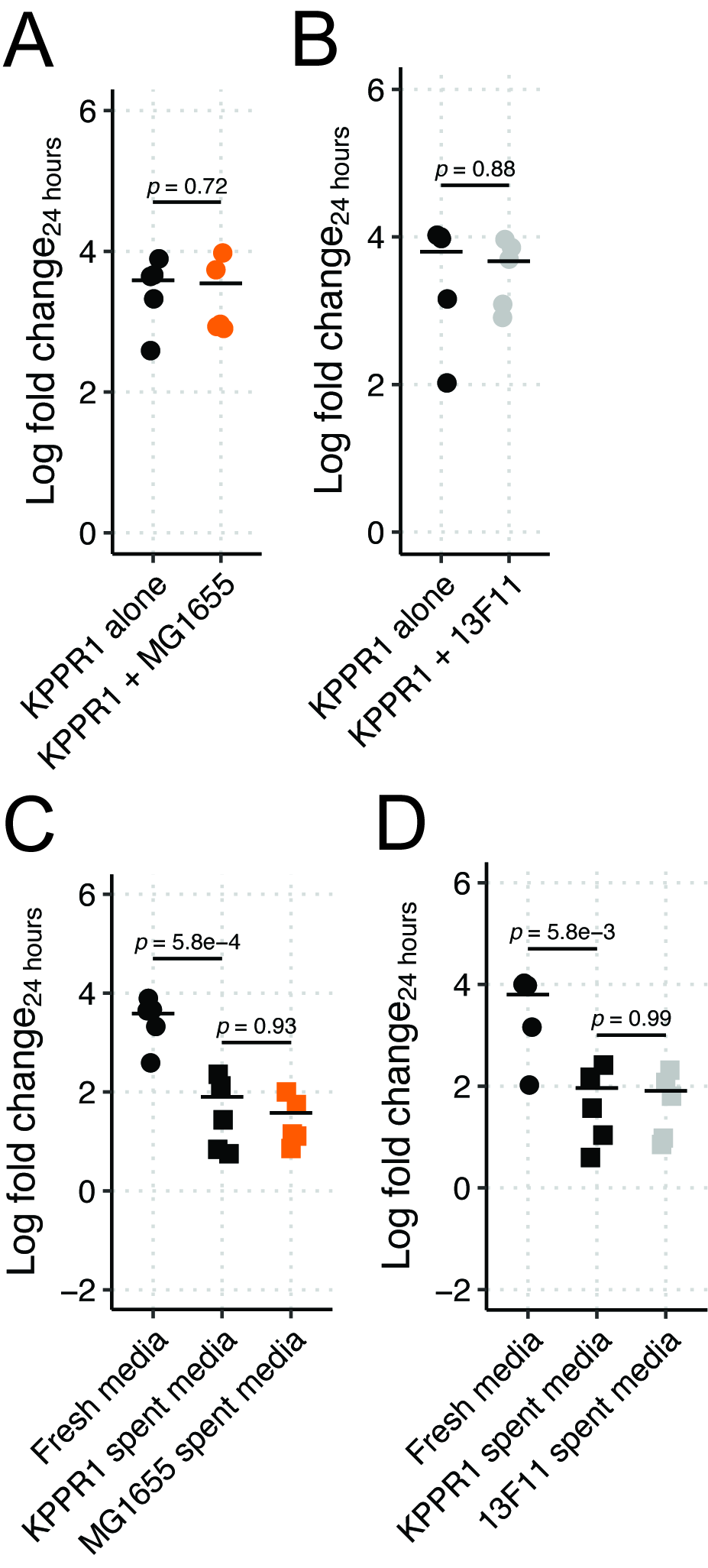

Supplement: S2 Fig — Kp KPPR1 was grown alone or in co-culture in LB with Ec MG1655 (A), Kp 13F11 (B), or in filter sterilized spent media of KPPR1, Ec MG1655 (C), or Kp 13F11 strain (D). For A–D, “Log fold change24 hours” = log10(output KPPR1 CFU at 24 hours/input KPPR1 CFU). p-values represent Tukey multiple comparison correction following one-way ANOVA. Each data point is a biological replicate, and horizontal lines indicate the mean of each dataset. The data underlying this Figure can be found in S2 Data. (TIF) [file pbio.3003809.s002.tif]

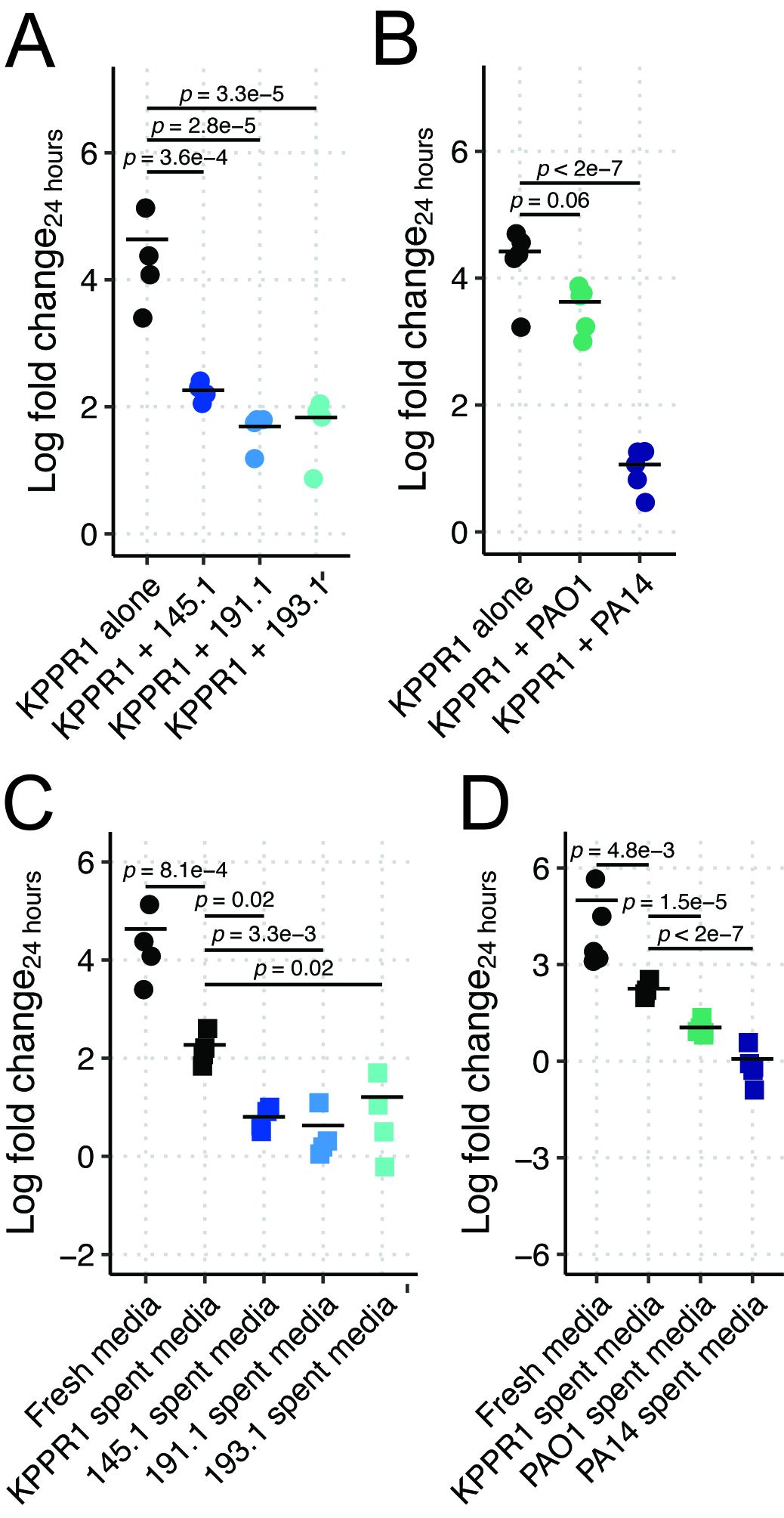

Supplement: S3 Fig — Kp KPPR1 was grown alone or in co-culture in M9 medium supplemented with 1.0% casamino acids with mouse-derived wild Pa (A), PAO1, PA14 (B) or in filter-sterilized spent media of KPPR1 or each Pa strain (C–D). For A–D, “Log fold change24 hours” = log10(output KPPR1 CFU at 24 hours/input KPPR1 CFU). p-values represent Tukey multiple comparison correction following one-way ANOVA. Each data point is a biological replicate, and horizontal lines indicate the mean of each dataset. The data underlying this Figure can be found in S2 Data. (TIF) [file pbio.3003809.s003.tif]

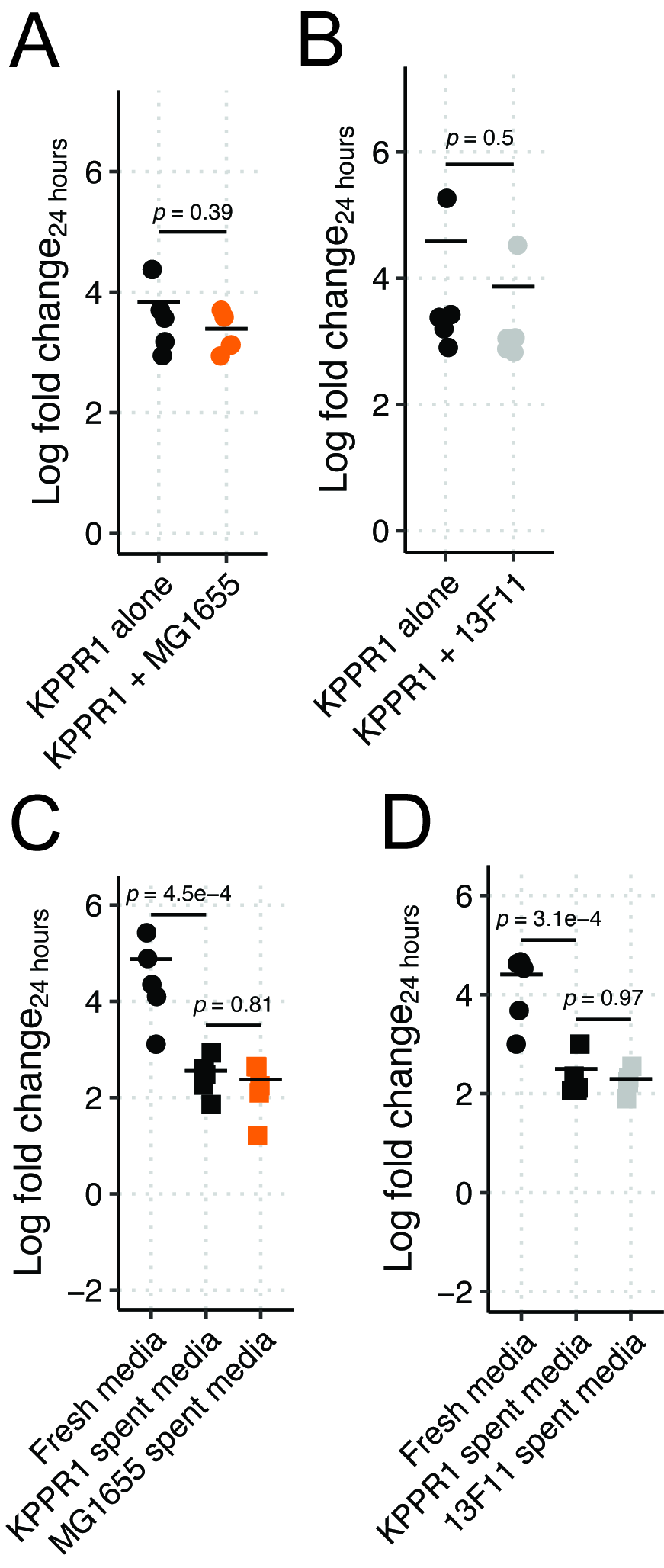

Supplement: S4 Fig — Kp KPPR1 was grown alone or in co-culture in M9 medium supplemented with 1.0% casamino acids with Ec MG1655 (A), Kp 13F11 (B), or in filter-sterilized spent media of KPPR1, Ec MG1655 (C), or Kp 13F11 strain (D). For A-D, “Log fold change24 hours” = log10(output KPPR1 CFU at 24 hours/input KPPR1 CFU). p-values represent Tukey multiple comparison correction following one-way ANOVA. Each data point is a biological replicate, and horizontal lines indicate the mean of each dataset. The data underlying this Figure can be found in S2 Data. (TIF) [file pbio.3003809.s004.tif]

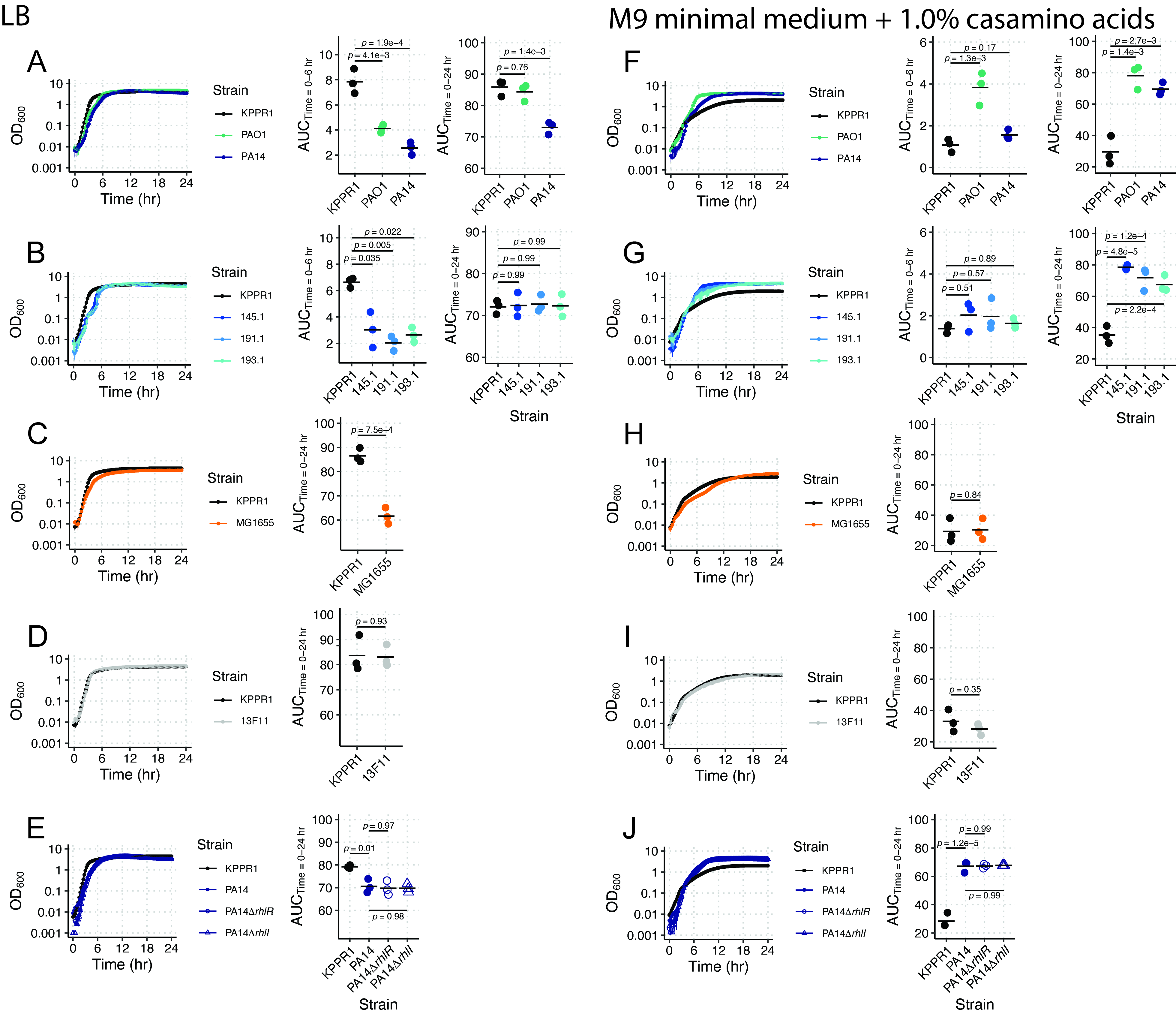

Supplement: S5 Fig — KPPR1, PAO1, PA14, Pa 145.1, Pa 191.1, Pa 193.1, MG1655, 13F11, PA14ΔrhlR, PA14ΔrhlI were grown in LB (A-E) or in M9 medium supplemented with 1.0% casamino acids (F-J). Area under the curve (AUC) analysis was used to quantify growth at early (0–6 hours, A-B, F-G) and late (0–24 hours, A-J) stages of growth. p-values represent Tukey multiple comparison correction following one-way ANOVA. For growth curves, each data point represents the mean, and vertical bars represent the standard error of the mean. For AUC analysis, each data point is a biological replicate, and horizontal lines indicate the mean of each dataset. The data underlying this Figure can be found in S2 Data. (TIF) [file pbio.3003809.s005.tif]

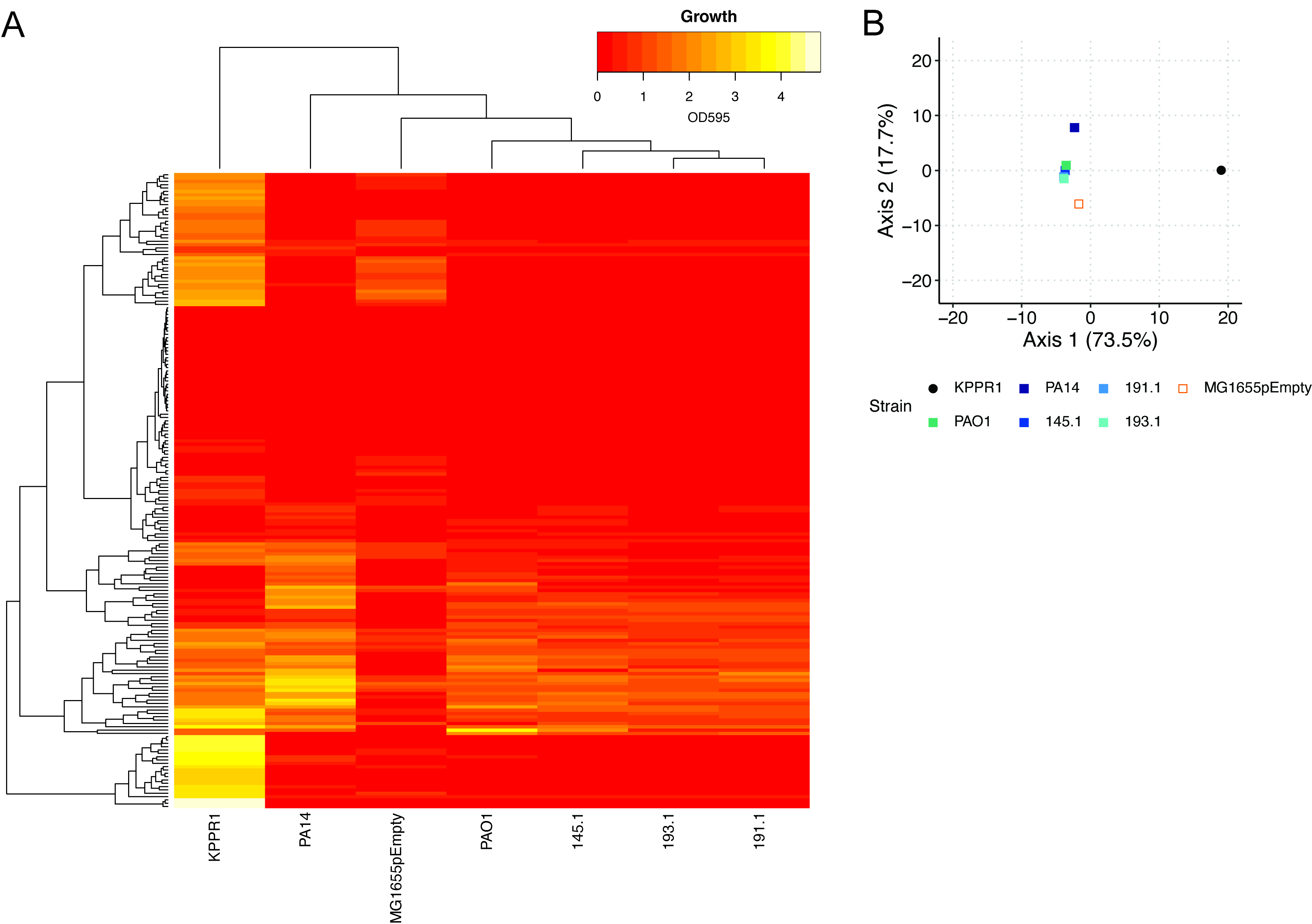

Supplement: S6 Fig — KPPR1, PAO1, PA14, Pa 145.1, Pa 191.1, Pa 193.1, and Ec MG1655pEmpty were grown in BioLog Phenotype Microarray plates PM1 and PM2 (mean of three biological replicates displayed, each row is an individual carbon source, A). Euclidean distance was used to measure the dissimilarity between the growth phenotypes of each strain (B). The data underlying this Figure can be found in S2 Data. (TIF) [file pbio.3003809.s006.tif]

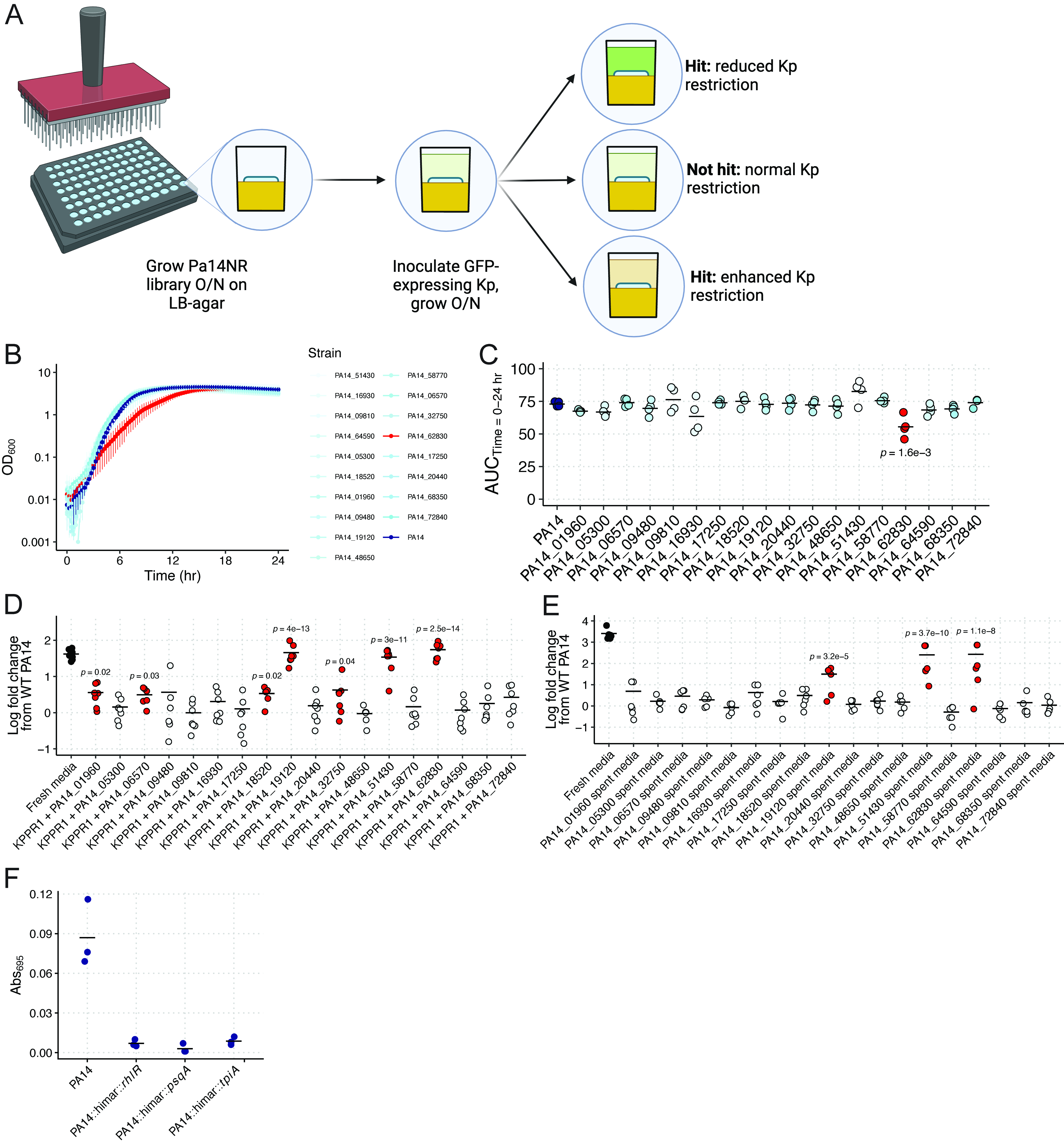

Supplement: S7 Fig — To identify candidate factors involved in Kp growth restriction, the PA14NR library was replicate plated onto LB-agar, grown overnight at 37 °C, and inoculated with GFP-expressing KPPR1 (A). After 24 hours of co-culture, the fluorescence of each co-culture was measured, yielding 18 candidate transposon mutants, 16 of which exhibited reduced restriction, and 2 that exhibited enhanced restriction. PA14 and the 18 candidate transposon mutants identified to have a role in Kp growth restriction were grown in LB (B) and area under the curve (AUC) analysis was used to quantify growth (C). In panel B, each data point represents the mean, and vertical bars represent the standard error of the mean. Kp KPPR1 was grown alone or in co-culture in LB with PA14 or the 18 transposon mutants (D) or in filter-sterilized spent media of KPPR1, PA14, or the 18 transposon mutants (E). PYO (F) and PYR (G) were measured at 695 and 500 nm, respectively, from select transposon mutants. For D–E, “Log fold change from WT PA14” = log10(output KPPR1 CFU at 24 hours/input KPPR1 CFU) in transposon mutant co-culture or spent media culture/ log10(output KPPR1 CFU at 24 hours/input KPPR1 CFU) in WT PA14 co-culture or spent media culture. For C, p-values represent Tukey multiple comparison correction following one-way ANOVA, and for D–E, p-values represent one-sample t test from a hypothetical mean of 0. For C–G, each data point is a biological replicate, horizontal lines indicate the mean of each dataset, and in C–E, red datasets are statistically significant from their relative comparisons. Panel A was Created in BioRender. Tilston-lunel, N. (2026) https://BioRender.com/6b5vwn8. The data underlying this Figure can be found in S2 Data. (TIF) [file pbio.3003809.s007.tif]

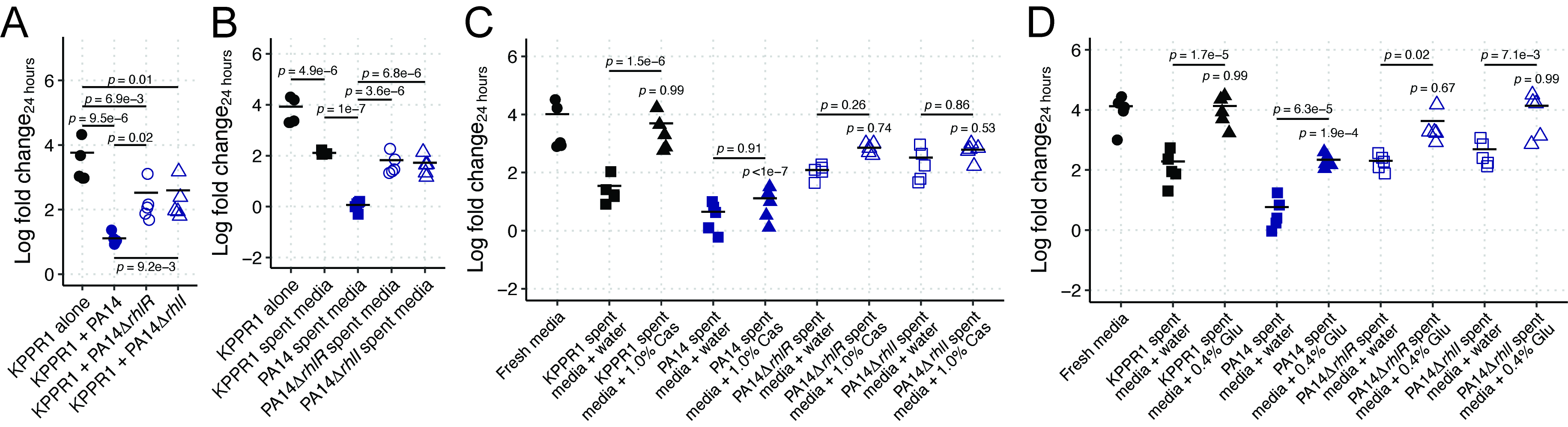

Supplement: S8 Fig — KPPR1 was grown alone or in co-culture in M9 medium supplemented with 1.0% casamino acids with WT PA14, PA14ΔrhlR, or PA14ΔrhlI (A) or in filter-sterilized spent media of KPPR1 or each Pa strain (B), supplemented with water, 1% casamino acids (“Cas,” C) or 0.4% glucose (“Glu,” D). For A-D, “Log fold change24 hours” = log10(output KPPR1 CFU at 24 hours/input KPPR1 CFU). p-values represent Tukey multiple comparison correction following one-way ANOVA. p-values over columns indicate comparison to “Fresh media” condition. Each data point is a biological replicate, and horizontal lines indicate the mean of each dataset. The data underlying this Figure can be found in S2 Data. (TIF) [file pbio.3003809.s008.tif]

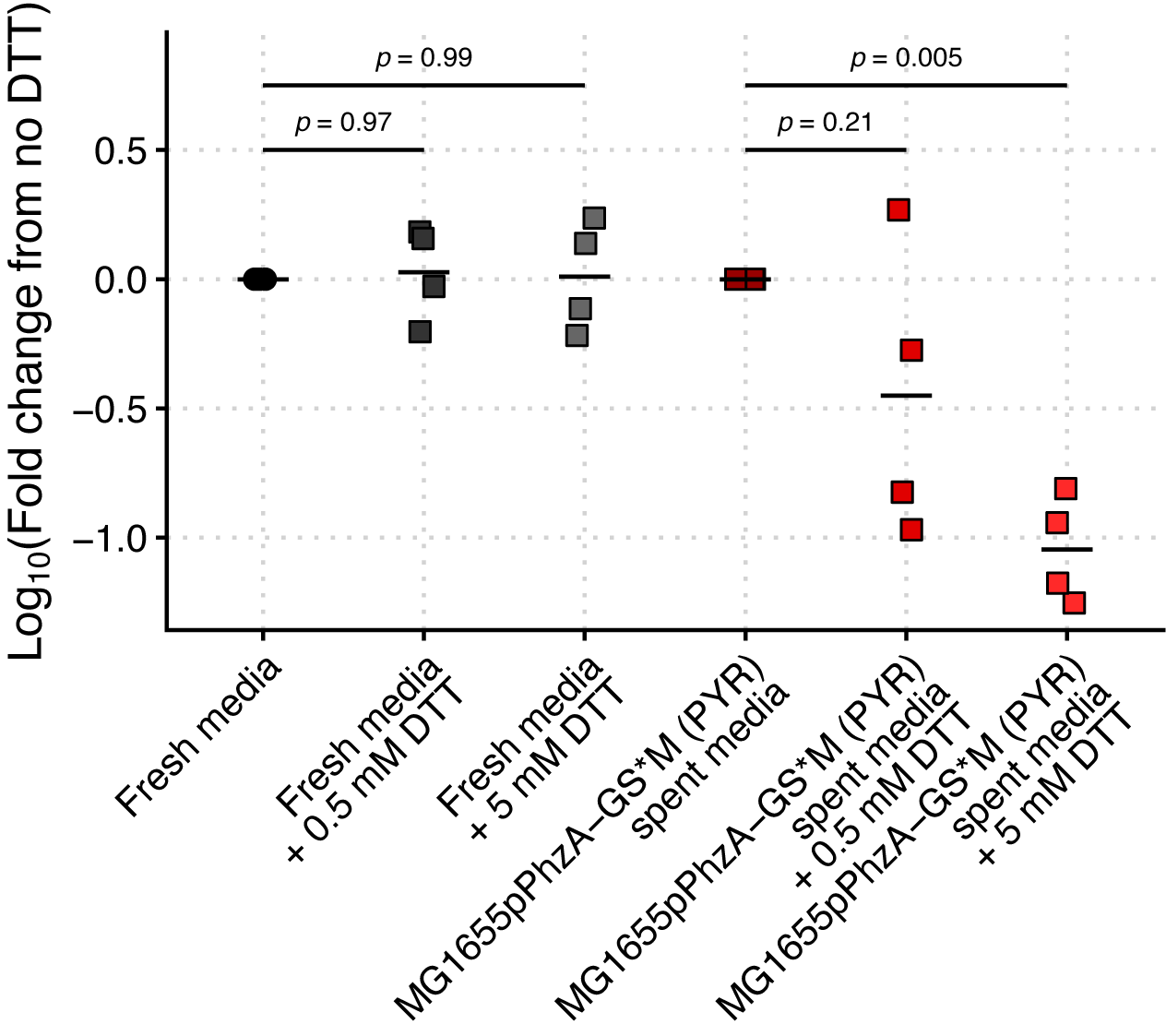

Supplement: S9 Fig — 13F11 (KanR KPPR1 variant) was grown in fresh LB broth or spent media from MG1655 constitutively expressing PYR (pPhzA-GS*M). Dithiothreitol (DTT) was titrated into both media. “Log10(Fold change from no DTT)” = log10(output 13F11 CFU at 24 hours/input 13F11 CFU) in fresh or spent media + DTT/ log10(output 13F11 CFU at 24 hours/input 13F11 CFU) in fresh or spent media without DTT. Each data point is a biological replicate, and horizontal lines indicate the mean of each dataset. The data underlying this Figure can be found in S2 Data. (TIF) [file pbio.3003809.s009.tif]

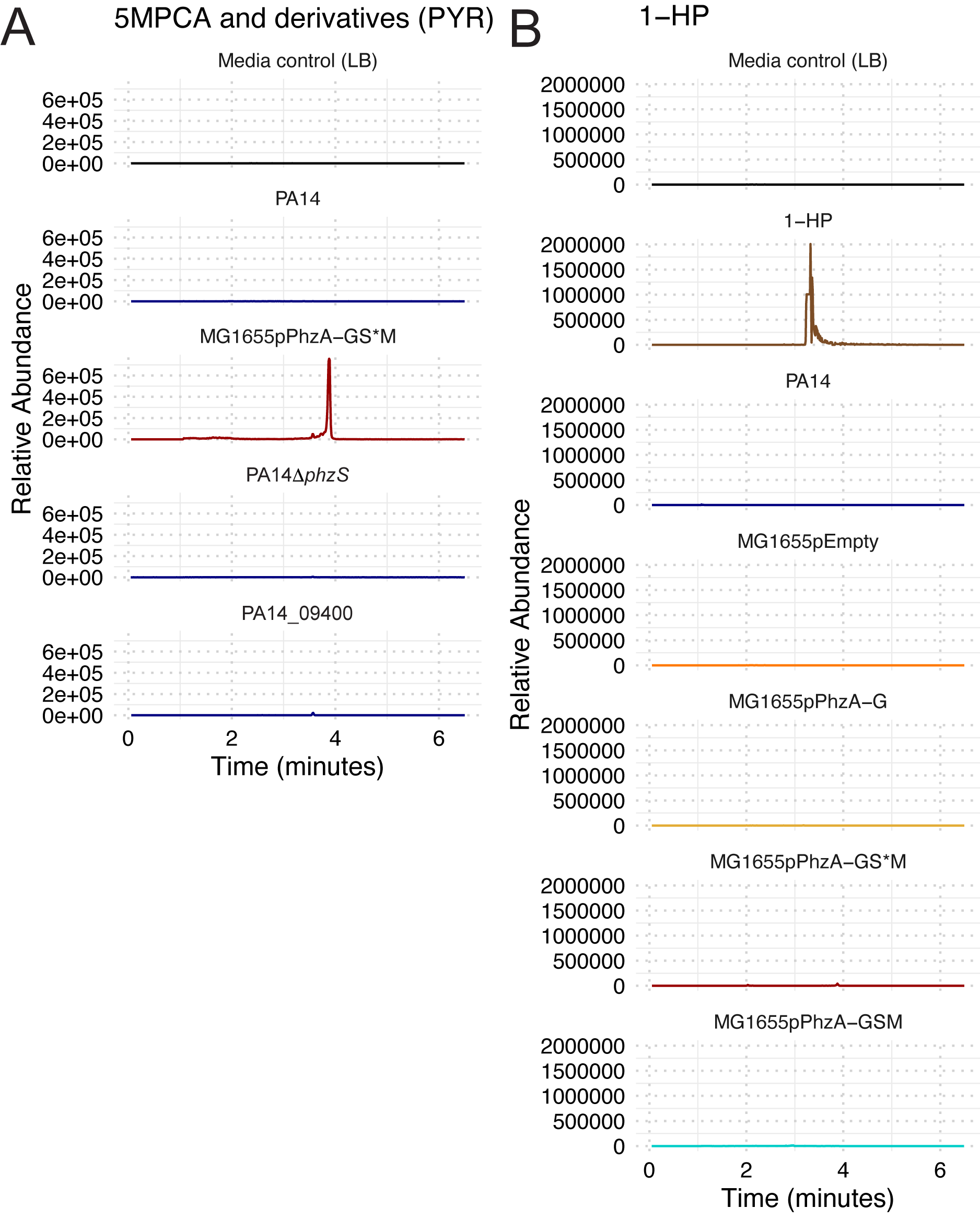

Supplement: S10 Fig — LC-MS was used to quantify PYR secretion from WT PA14, MG1655pPhzA-GS*M, PA14ΔphzS, and PA14_09400, which is a phzS transposon mutant from PA14NR library (A) or 1-HP secretion from WT PA14, MG1655pEmpty, MG1655pPhzA-G, MG1655pPhzA-GS*M, or MG1655pPhzA-GSM in comparison to pure 1-HP (B). The data underlying this Figure can be found in S2 Data. (TIF) [file pbio.3003809.s010.tif]

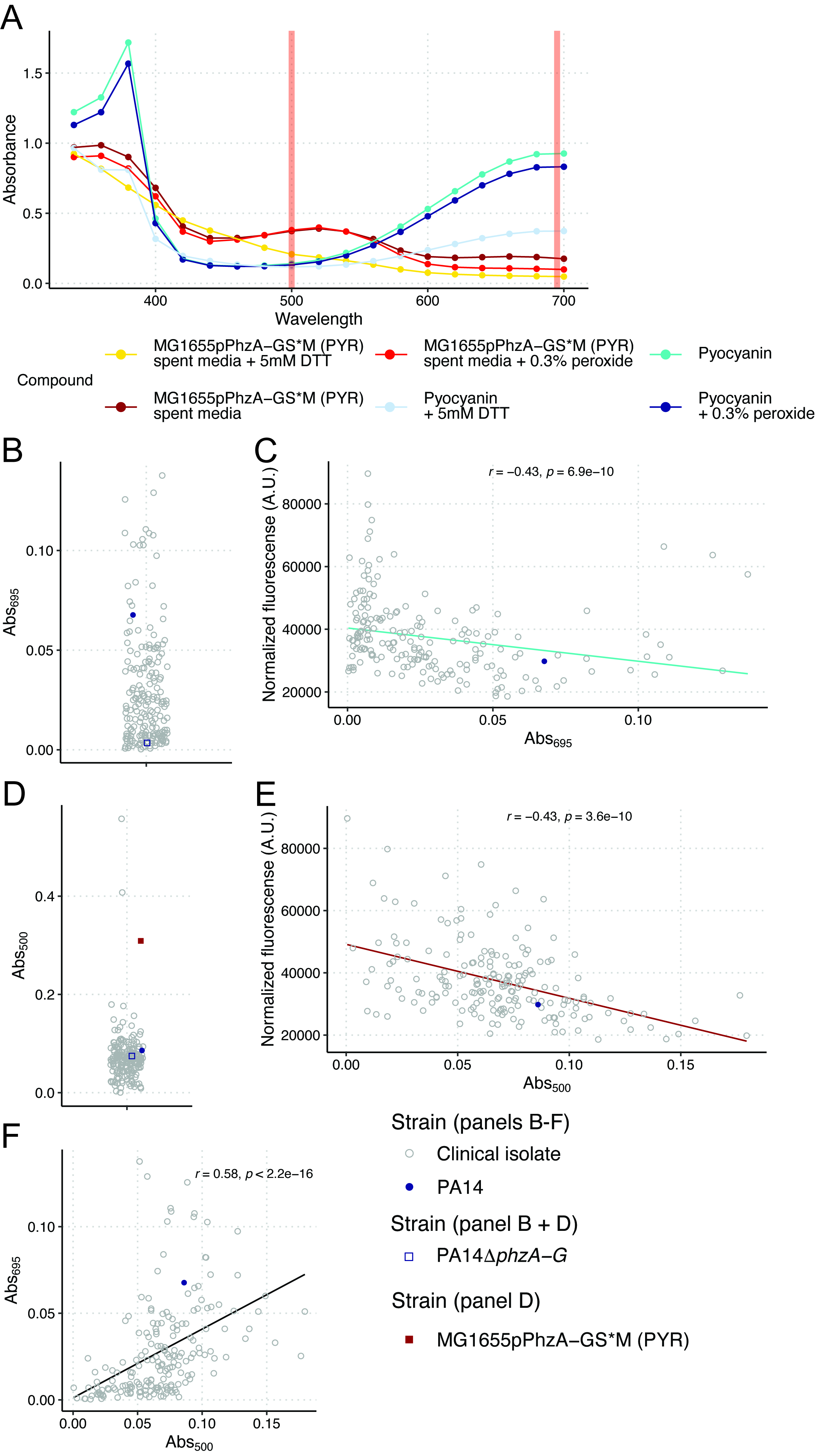

Supplement: S11 Fig — The spectra of pure PYO and the spent media MG1655 constitutively expressing PYR (pPhzA-GS*M) was measured under native, oxidizing (0.3% hydrogen peroxide) and reducing (5 mM dithiothreitol [DTT]) conditions (A) to identify wavelengths at which those phenazines can be differentiated. We determined that PYO and PYR in oxidizing conditions can be differentiated at 695 and 500 nm, respectively (red vertical bars). Clinical Pa strains (N = 194), WT PA14, and PA14ΔphzA-G were grown in LB broth and the Abs695 of spent media was measured after 24 hours (B). Abs695 results were correlated to growth restriction results from Fig 5C (Spearman correlation test, C). Clinical Pa strains (N = 192), WT PA14, PA14ΔphzA-G, and MG1655 constitutively expressing PYR (pPhzA-GS*M) and PYO (pPhzA-GSM) were grown in LB broth and the Abs500 of spent media was measured after 24 hours following oxidation by 0.3% hydrogen peroxide (D). Abs500 results were correlated to growth restriction results from Fig 5C (Spearman correlation test, E). Abs500 and Abs695 results were correlated with one another (Spearman correlation test, F). Each data point represents the mean read of each culture. The data underlying this Figure can be found in S2 Data. (TIF) [file pbio.3003809.s011.tif]

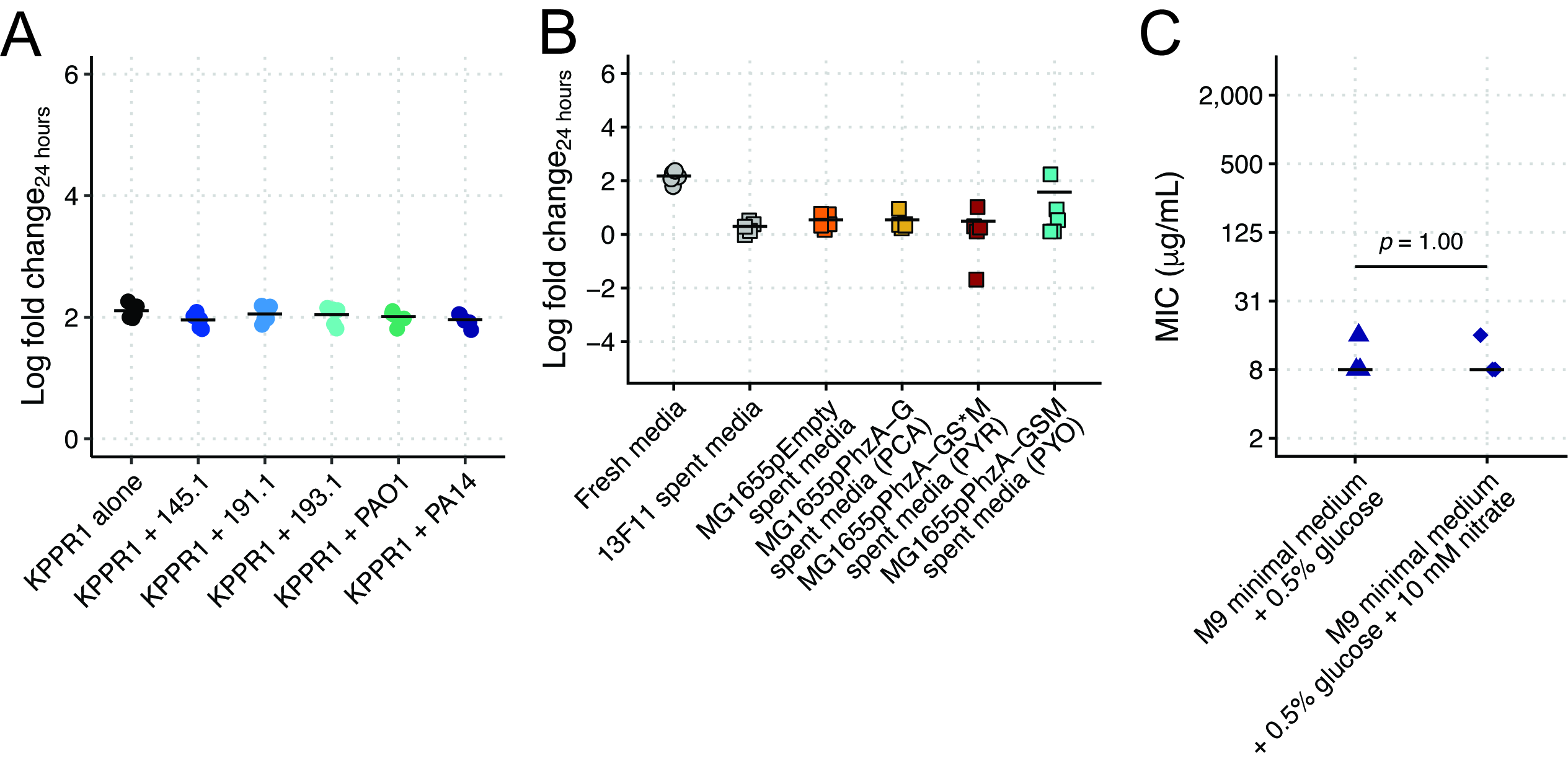

Supplement: S12 Fig — KPPR1 was grown anaerobically alone or in co-culture in LB with mouse-derived wild Pa, PAO1, and PA14 (A). 13F11 (KanR KPPR1 variant) was grown anaerobically in filter-sterilized spent media of MG1655 containing an empty vector (pEmpty) or constitutively expressing PCA (pPhzA-G), PYR (pPhzA-GS*M), and PYO (pPhzA-GSM, B). The minimum inhibitory concentration (MIC) and minimum bactericidal concentrations (MBC) were determined for KPPR1 for pure PYO in M9 minimal media with 0.5% glucose and 10 mM NaNO3 (C). For A-B, “Log fold change24 hours” = log10(output Kp CFU at 24 hours/input Kp CFU). Each data point is a biological replicate; horizontal lines indicate the mean of each dataset. For C, p-values represent Tukey multiple comparison correction following one-way ANOVA, comparing aerobic versus anaerobic MICs within each phenazine. Note that 13F11 growth in MG1655 spent media is no different than self-spent media in anaerobic conditions, whereas growth is restricted in the presence of PYR and PYO in aerobic conditions (see Fig 3F). The data underlying this Figure can be found in S2 Data. (TIF) [file pbio.3003809.s012.tif]

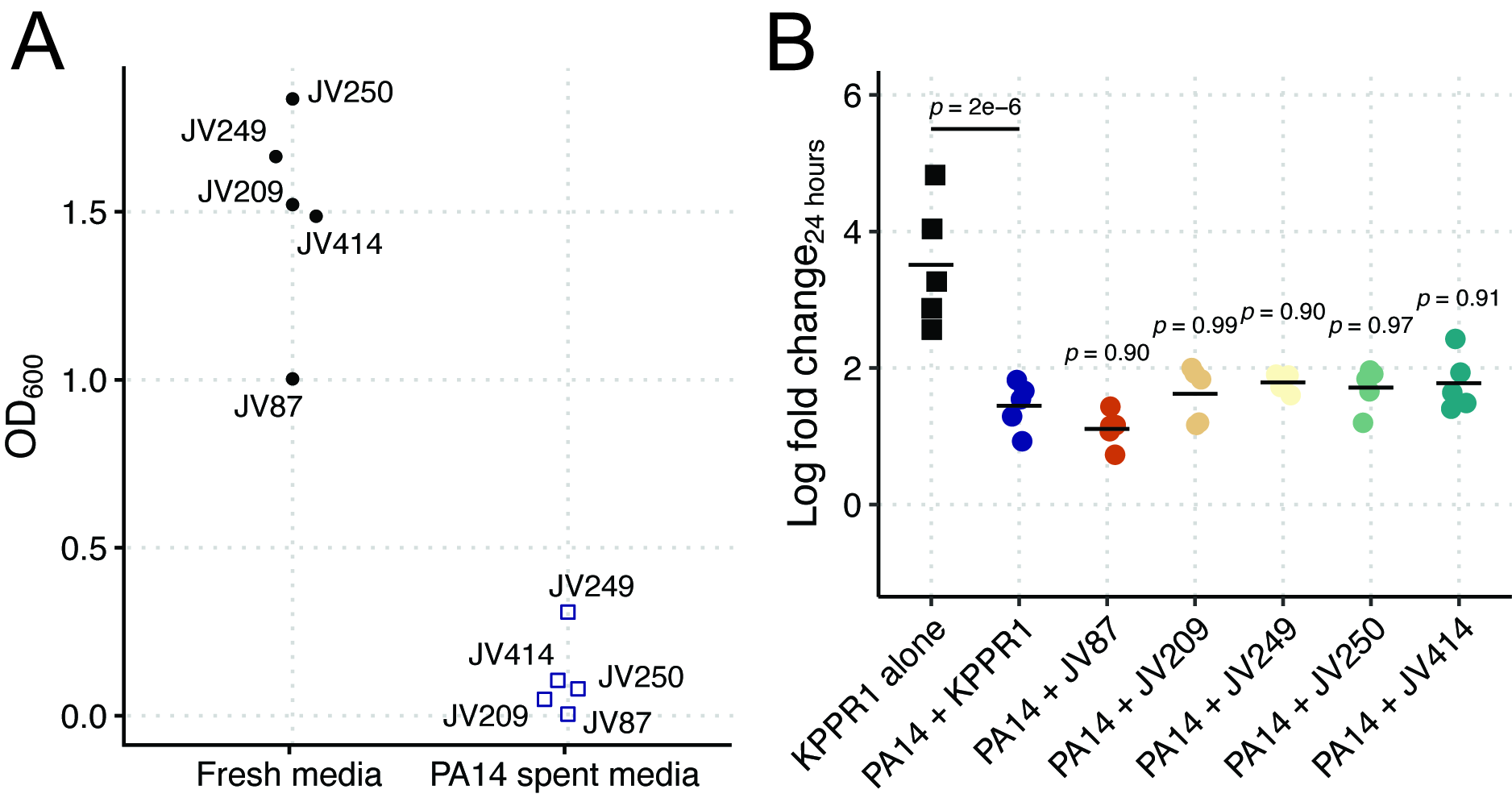

Supplement: S13 Fig — Clinical Kp strains (N = 6) were selected for validation (A). KPPR1 or select Kp strains were grown alone or co-cultured with select clinical Pa strains (B). For B, “Log fold change24 hours” = log10(output KPPR1 CFU at 24 hours/input KPPR1 CFU). p-values represent Tukey multiple comparison correction following one-way ANOVA compared to the “PA14 + KPPR1” condition. Each data point is a biological replicate, and horizontal lines indicate the mean of each dataset. The data underlying this Figure can be found in S2 Data. (TIF) [file pbio.3003809.s013.tif]

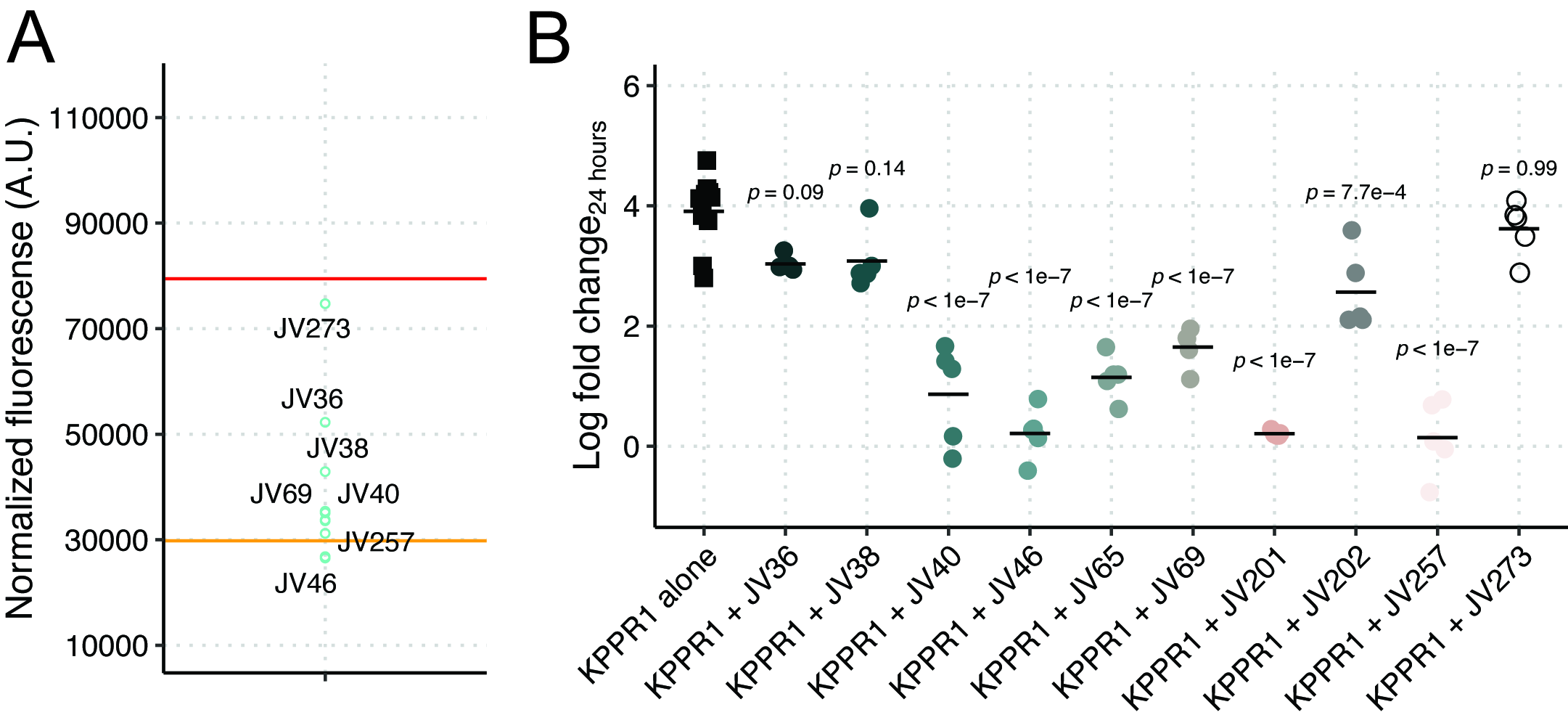

Supplement: S14 Fig — Clinical Pa strains (N = 10) were selected for validation (A). KPPR1 was grown alone or in co-culture with select clinical Pa strains (B). For B, “Log fold change24 hours” = log10(output KPPR1 CFU at 24 hours/input KPPR1 CFU). p-values represent Tukey multiple comparison correction following one-way ANOVA compared to the “KPPR1 alone” condition. Each data point is a biological replicate, and horizontal lines indicate the mean of each dataset. The red line represents the mean normalized fluorescence for KPPR1, and the orange line represents the mean normalized fluorescence for KPPR1 + PA14. The data underlying this Figure can be found in S2 Data. (TIF) [file pbio.3003809.s014.tif]

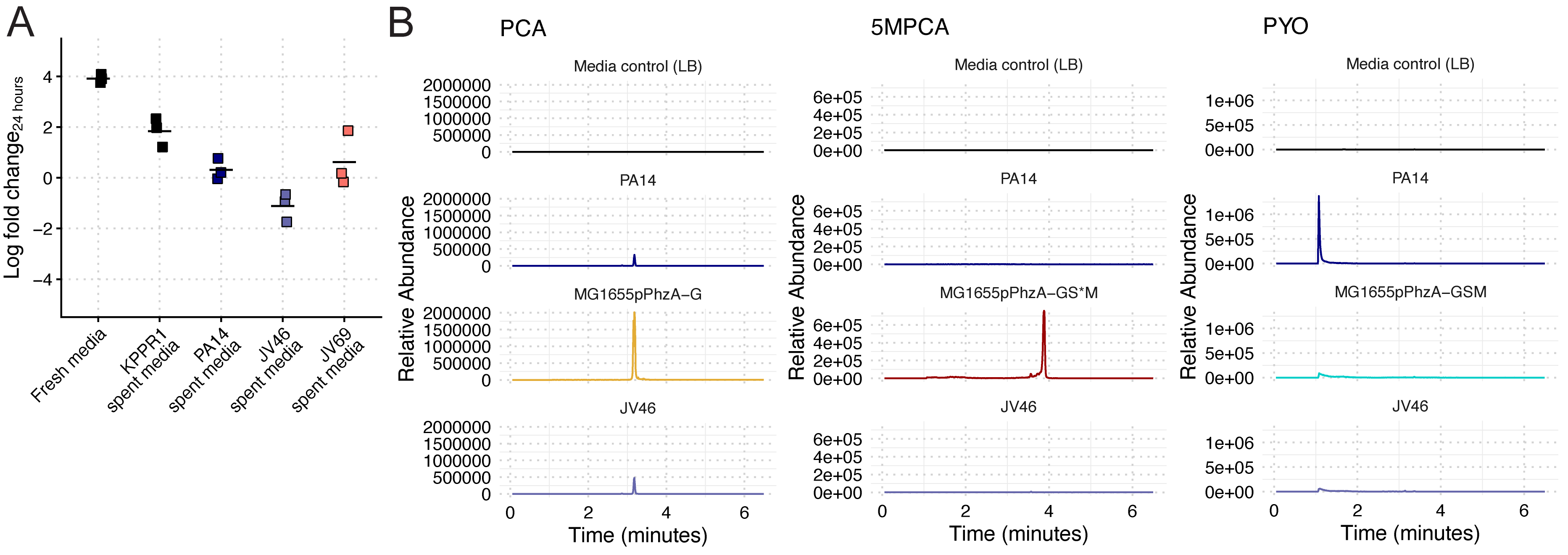

Supplement: S15 Fig — KPPR1 was grown in fresh LB medium or filter-sterilized spent media of KPPR1, PA14, JV46, or JV69 (A). “Log fold change24 hours” = log10(output KPPR1 CFU at 24 hours/input KPPR1 CFU). Datapoints outlined in red are below the limit of detection (200 CFU/mL). p-values represent Tukey multiple comparison correction following one-way ANOVA. Each data point is a biological replicate, and horizontal lines indicate the mean of each dataset. LC-MS was used to quantify phenazine secretion from JV46 (B). The data underlying this Figure can be found in S2 Data. (TIF) [file pbio.3003809.s015.tif]

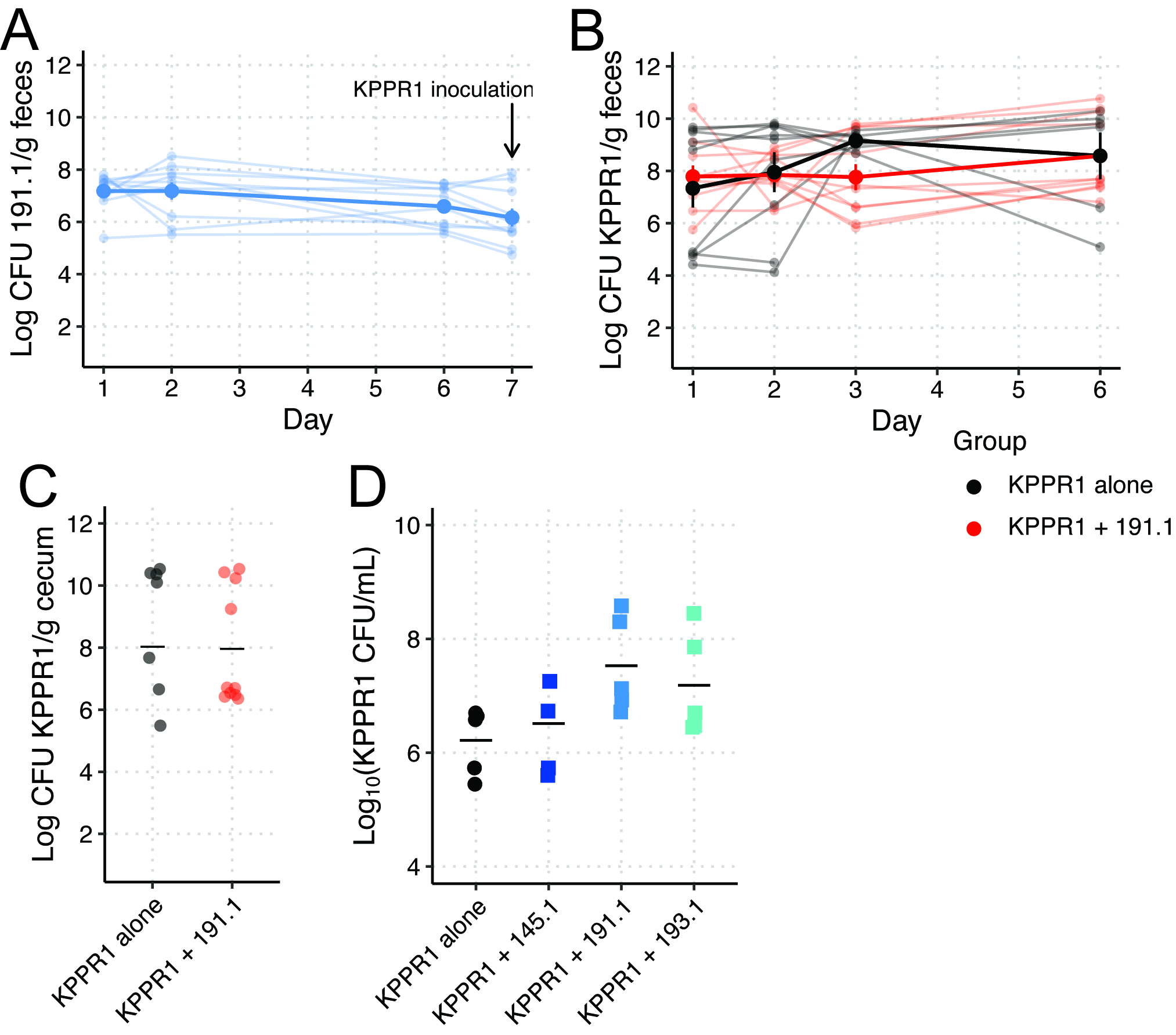

Supplement: S16 Fig — C57Bl6/J mice were treated for 4 days with 0.5 g/L ampicillin, then orally gavaged with ~107 CFU 191.1. Seven days post-191.1 colonization, mice were orally gavaged a second time with ~108 CFU KPPR1 (N = 10). Antibiotic-treated KPPR1 mono-colonized mice served as a control (N = 10). 191.1 fecal loads were monitored post-colonization (A), and KPPR1 fecal (B) and cecal loads (C) were measured were monitored post-colonization (after 7 days 191.1 colonization) or at the end of the experiment, respectively. Large intestinal contents from C57Bl6/J (no antibiotic treatment, N = 5) mice were collected and resuspended in sterile PBS. ~5 × 10^7^ CFU KPPR1 alone or an equal ratio of KPPR1 with each wild Pa, was inoculated into large intestinal contents and grown anaerobically. KPPR1 density was measured at 48 hours (D). The data underlying this Figure can be found in S2 Data. (TIF) [file pbio.3003809.s016.tif]

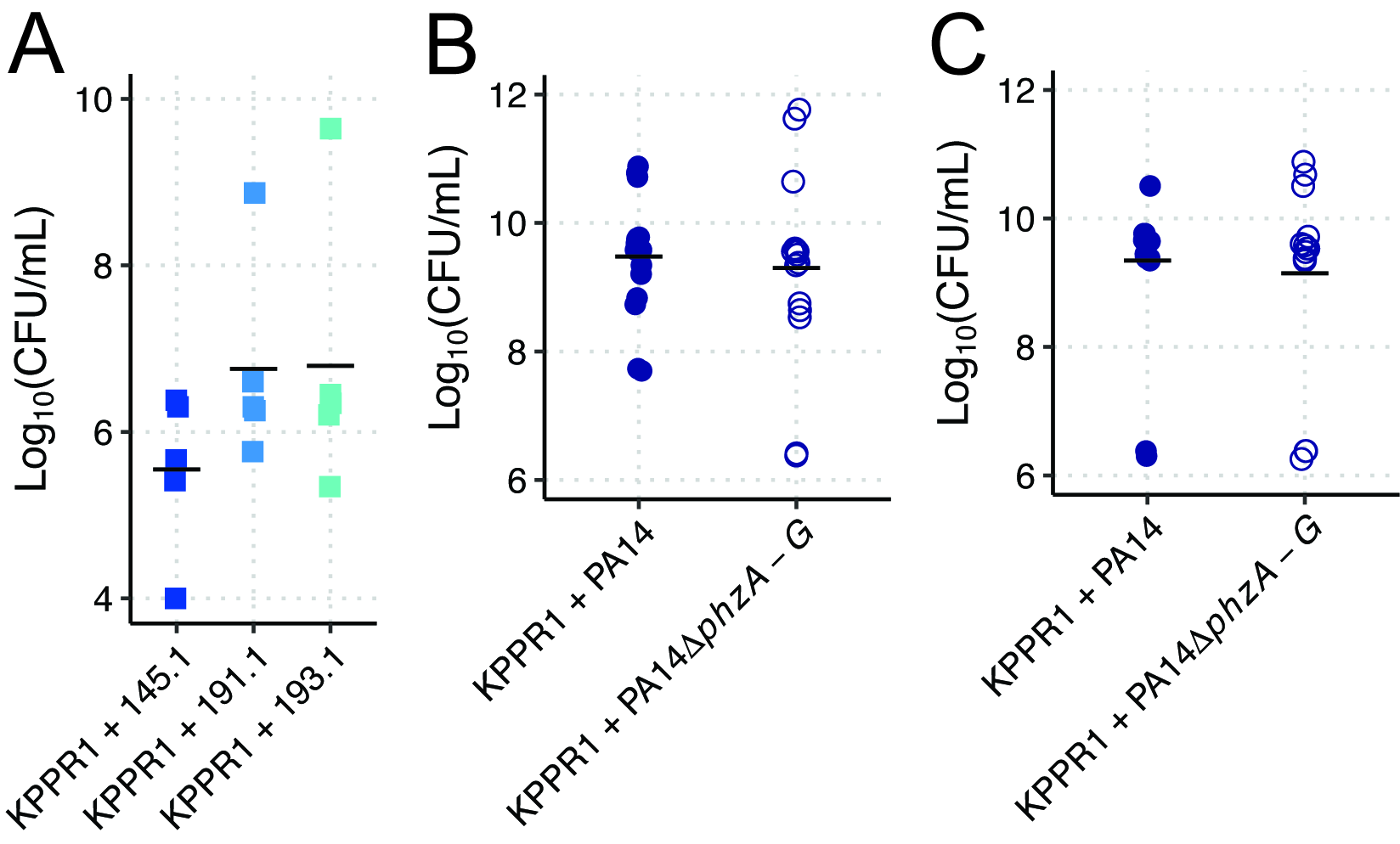

Supplement: S17 Fig — Pa density was measured in large intestinal contents (A), BALF (B), and bladder homogenate (C) from experiments presented in Figs S15A, 6B, and 6C, respectively. The data underlying this Figure can be found in S2 Data. (TIF) [file pbio.3003809.s017.tif]
