# Supplementary material for: Environmental redox conditions and strain variation define phenazine-mediated antagonism in co-infecting bacteria
Source: PLoS Biol. 2026 May 20;24(5):e3003809. doi: 10.1371/journal.pbio.3003809 (PMC13197074; doi:10.1371/journal.pbio.3003809)
Supplement: Attachment — Submitted filename: Response to reviewers.docx [file pbio.3003809.s023.docx]

**Response to reviewers**

*We thank the reviewers for their time and thoughtfulness in assessing our manuscript. We have carefully considered the reviewers’ comments and concerns, and we believe that our manuscript is now significantly stronger. Our responses to the reviewers’ comments and concerns are in blue italics and annotated throughout the marked-up manuscript document using track changes.*

**Reviewer #1:**

This manuscript by Todd *et al* describes a novel interaction between *P. aeruginosa* isolates and *Klebsiella* isolates, in which Pa restricts the growth of Kp under aerobic conditions. The authors lead the reader through an initial observation from an unrelated mouse study in which oral gavage with Kp failed to establish gut colonization in mice, and sequencing of potential culprits revealed three Pa isolates capable of restricting Kp growth. The majority of the manuscript is devoted to probing the underlying mechanism, through which the authors discover that the growth-restricting factor is secreted and that restriction can be influenced by nutrient and oxygen availability. Through screening a transposon mutant library of a Pa strain, the authors determined that growth inhibition was likely due to phenazine production and regulation of production by quorum sensing. Through testing a series of Pa mutants and Ec heterologous expression constructs, the authors conclude that Pa largely restricts Kp growth through production of pyocyanin and pyorubin. Despite the initial mouse growth restriction data that prompted this study, colonization of mice by Pa failed to restrict Kp growth *in vivo* or in large intestinal content *ex vivo*. The authors conclude the body of work by demonstrating that a wide array of *Klebsiella* isolates and two E. coli isolates are restricted by phenazines, but Pa isolates vary widely in ability to restrict Kp growth. They also found that Pa isolates from different body sites differ in restrictive ability, with those from blood and urine being the most restrictive. Taken together, the authors state that these data provide evidence for environment-dependent modulation of pathogen interactions.

This is an enormous body of work and provides further support for considering the genomic and environmental context of bacterial isolates with examining pathogen interactions. There are also several notable strengths, such as the assessment of strain-specific differences and directly quantifying PYO production by the clinical isolates and Pa strains and reconciling these data with the MIC for PYO against Kp. The manuscript is well-written overall, although there are some areas in the results section that are difficult to follow. The main weakness of the study is that the majority of the conclusions regarding the specific contributions of phenazines to growth restriction are only examined using spent media, without confirmation using purchased/purified PCA, PYO, 1-HP, etc. Another key weakness is the lack of Pa-mediated growth restriction in the mouse studies, although the authors address potential reasons for this discrepancy in the discussion. Further comments for consideration are provided below.

*We thank the reviewer for their thorough evaluation of our manuscript and positive feedback. We agree with the reviewer’s suggestions for improving our study and have outlined our changes and responses to weaknesses below.*

Major Points:

1.      PCA, PYO, and 1-HP can all be purchased, yet the methods indicate that only PYO was purchased and it was only used for the MIC/MBC experiments. Direct confirmation of the ability of each chemical to restrict Kp growth in fresh media compared to spent media and under aerobic vs anaerobic conditions would substantially increase impact of the study and confidence in the conclusions.

*We agree this would strengthen the study; we therefore purchased PCA and 1-HP and performed the requested assays and confirmed that 1) PYO is more active than PCA and 1-HP, as noted by higher MICs and 2) these differences were observed in both fresh and spent media. A technical note on these experiments: we are not confident that we have determined the exact MIC for PCA, as its solubility in aqueous solvents is somewhat limited compared to PYO and 1-HP. The apparent PCA MIC was likely affected by the large amount of DMSO in culture media used for this condition. DMSO restricts Kp growth at ~10%, and the culture medium at which the MIC was observed contains 12.5% DMSO. Nonetheless, we are confident that PYO is more bioactive than PCA, as this technical issue would lead to an overestimation of the bioactivity of PCA. These data in Figure 4, and we have added additional text describing these results, which is below:*

*Next, we aimed to assess the antimicrobial activity of phenazines against KPPR1. First, we measured the minimum inhibitory (MIC) and minimum bactericidal concentrations (MBC) of pure PYO, as it was unclear from our co-culture approach if this compound was bacteriostatic or bactericidal. To replicate our co-culture and spent media culture assays, we measured the MIC and MBC of PYO. We performed these assays in both fresh LB and PA14ΔphzA-G spent LB, the latter of which we used to mimic the nutrient conditions in which Kp is experiencing phenazine-induced stress, as nutrient availability will modulate phenazine sensitivity by changing the cellular redox state [54]. The MIC of PYO was 8- and 32-fold lower than the MBC (Figure 4A-B), in fresh and spent media, respectively, indicating that PYO is primarily bacteriostatic, which is consistent with the ability of PYO to arrest bacterial respiration [46], as opposed to kanamycin, which has an MIC only 2-fold lower than the MBC, indicating bactericidal activity (Figure 4A-B). Then, we sought to confirm our co-culture data that demonstrated that PCA and 1-HP had no activity against KPPR1 (Figure 3D). The MICs of both compounds in pure form were higher than that of PYO both fresh (Figure 4C) and spent (Figure 4D) media. Of note, the calculated PCA MIC is likely an overestimation of activity, as the large amount of DMSO (>10% volume at ≥250 μg/mL PCA) in culture medium used to maintain PCA solubility likely affected KPPR1 growth. Nonetheless, these data support our findings that PYO restricts Kp growth, whereas PCA and 1-HP have little or no effect.*

*Summary of changes: Addition of MIC data to Figure 4 and corresponding text.*

2.      In the discussion, the authors hypothesize that "environmental redox gates the potency of phenazine-mediated interference." This could be tested fairly easily by examining whether Kp growth is more highly restricted by purchased/purified phenazines under aerobic vs anaerobic conditions, and the results of such an experiment would increase the strength of the conclusions and overall impact of the body of work.

*This is another good suggestion. We have performed these experiments and confirmed that the bioactivity of PYO was reduced under anaerobic conditions, as noted by a higher MIC in anaerobic conditions. We have also tested the MIC of PCA and 1-HP under anaerobic conditions, but noted no substantial changes, potentially due to their already low potency. These data are in Figure 4, and we have added additional text describing these results, which is below:*

*Some studies have shown that PYO remains active under anoxic conditions [55-57], indicating that the cytotoxicity of phenazines is not solely dependent on aerobic respiration. To determine if this is the case for KPPR1, we also measured the MIC of PCA, 1-HP, and PYO under anaerobic conditions. Unlike prior studies, we observed a significant reduction of PYO activity in anaerobic conditions in both fresh (Figure 4C) and spent (Figure 4D) media.*

*Summary of changes: Addition of MIC data to Figure 4 and corresponding text.*

3.      With respect to the LC-MS data, running purified PCA, PYO, and 1-HP as a standard would greatly increase confidence in the results. This is especially important as the peak that is assumed to be PCA for MG1655pPhzA-G eluted at a different time that the peak for PA14. Can any further information be provided to demonstrate level of confidence that this peak represents PCA, or whether the PCA produced by E. coli may differ in some way from that produced by PA14?

*This is an excellent and fruitful suggestion. First, we did not detect 1-HP in the spent media of any of our samples, including WT PA14. Despite a negative finding, we decided to include these data as there are other studies that report 1-HP production from WT PA14, although these studies used different Pa culture approaches. These data are in Figure S10B, and text has been added to the manuscript describing these results:*

*Finally, we did not detect 1-HP secretion from any of our Pa or Ec strains (Figure S10B).*

*Second, in running the PCA standard, we realized that the PCA undergoes significant fragmentation. Thus, the data we reported in the original manuscript corresponds to a mass that is not appropriate for measuring PCA (m/z 225). Upon re-analysis with the more appropriate mass (m/z 207), we show that indeed, the PCA produced by our Ec strain corresponds to that of PA14. We have updated the data in Figure 3G and S15B with the appropriate PCA plots.*

*When we ran phenazine standards, we observed a modest retention-time shift consistent with column aging between runs (~1 year). To be transparent, we provide the PYO and PCA standards as a reviewer-only figure (****Figure R1****). Ideally, we would compare the change in retention time to PYO; however, this is challenging because the PYO retention time is very close to the start of data collection. Moreover, further optimization of the PYO we load on the column would be required to get the necessary resolution to detect age-related column drift. Given that 1) the exact masses of the PCA standard and peaks shown in Figure 3G match and 2) the retention times at this mass align for our Pa and Ec datasets, we believe that our MG1655pPhzA-G strain is producing PCA.*

***Figure R1.*** *LC-MS was used to quantify phenazine secretion from Ec and Pa strains compared to PCA (****A****) and PYO (****B****) standards.*

*Summary of changes: Addition of LC-MS data to Figure S10B, adjustment of LC-MS data in Figure 3, and changes to corresponding text.*

4.      It is interesting that the supernatant from the PYR over-expressing strain was bactericidal, yet the PA14phzS mutant did not restrict growth of Kp, considering that inability to convert 5MPCA to PYO would be expected to result in accumulation of 5MPCA and PYR. Further comment on this observation is warranted in the discussion, particularly if it may be due to the unusual peaks in the PYO LCMS for this strain that are absent in the other strains.

*We agree this is an important point. We originally performed LC-MS for the phzS mutant strains, and it appears that the PYR peak is much lower, and thus, was insufficient to inhibit KPPR1 growth. We did not include this in the original manuscript but now include it. A potential explanation for this finding is that the PhzM and PhzS form a complex that allows for rapid conversion of 5MPCA to PYO, which may stabilize the proteins in a way that facilitates activity. Thus, it is possible that in our MG1655 over-expression strain, PhzS is intact but mutated, whereas our PA14 phzS mutants cannot form such a complex. Alternatively, it may be that PA14 is rapidly modulating PYR to avoid any toxicity; however, these possibilities are not distinguishable with the current dataset and would require much more extensive experimentation to confirm. We have added the LC-MS data to the supplement (Figure S10A), and additional text describing the results, which is below:*

*Given that mutation of PhzS led to PYR overexpression in our Ec system, we were curious as to why our PA14 phzS mutant was not equally inhibitory (Figure 3D-F), as this mutant has the genes necessary to produce PYR. We performed high-resolution mass spectrometry of the spent media from this mutant, as well as a phzS transposon mutant from our PA14 transposon library screen. We did not detect substantial PYR levels from these strains (Figure S10A), suggesting that at least partially intact PhzS is required to produce PYR or that PA14 has alternative pathways to modify PYR.*

*Summary of changes: Addition of LC-MS data to Figure and changes to corresponding text.*

5.      All of the data are reported as log fold change in CFUs at experimental endpoint versus the input. While it does allow all data to be compared even if there are day-to-day changes in starting inoculum, it would useful to show the actual CFU values for key experiments, at least as supplemental or supporting information.

*All CFU data are present in the raw data, which is referenced in our data availability statement. We have modified the data availability statement to directly indicate this and have included the raw data files as part of the reviewer materials. Additionally, we have lifted the embargo on this dataset to allow for review. The additional text is below:*

*All raw data, including all CFU/mL values and analysis scripts used for this study are available at* [*https://github.com/jayvorn/Pseudomonas-aeruginosa-and-Klebsiella-pneumoniae-phenazines*](https://github.com/jayvorn/Pseudomonas-aeruginosa-and-Klebsiella-pneumoniae-phenazines)*.*

6.      It was unclear from the body of the manuscript if the GS*M was a known/existing mutation or spontaneously created in this study. Upon re-reading the methods section, it appears to have been selected based on pigment production. I recommend providing additional rationale for the approach that was used, as well as sequencing the E. coli strain harboring the GS*M plasmid and providing a genome comparison to E. coli with either the empty vector or the GSM plasmid to confirm that no other changes have occurred in this strain that may impact Kp growth.

*We apologize for the lack of clarity, which has been rectified in the amended methods section. The phzA-GS*M was spontaneously identified in this study. PYO is cytotoxic to E. coli, which leads to instability in the phzA-GSM mutant. Serial plating of the mutant led to the emergence of red-pigmented colonies, with plasmid sequencing of one such colony identifying the phzS mutation. This plasmid was subsequently purified and re-transformed into a fresh MG1655 E. coli WT to ensure the plasmid alone was responsible for the PYR production, and any related growth restriction of Pa. The text added to the methods section are below:*

*The PYR expression plasmid (pSB3K3_phzA-GS*M) was obtained spontaneously by serially plating on LB-agar, leading to the identification of a colony which displayed a red corona. The plasmid was subsequently purified and re-transformed into a fresh E. coli SJ102 background strain to avoid any genomic mutations.*

*Additionally, we sequenced the phzA-GS*M and phzA-GSM strains using our in-lab MinION to determine if there were any obvious mutations in the phzA-GS*M chromosome compared to the reference MG1655 chromosome (NC_000913) or the phzA-GSM strain. We found that the phzA-GS*M and phzA-GSM strains shared six mutations, of which, four were detected in >50% of reads, suggesting that these mutations originated in the parental MG1655 clone used for these experiments. We identified five and three mutations unique to the phzA-GS*M and phzA-GSM strains, respectively; however, none of these mutations were detected in >50% of reads. The low prevalence of these reads suggest that these are likely a result of the baseline error rate of our sequencing approach (FLO-MIN114 flow cell with real-time basecalling, max error rate of ~0.5%). Moreover, 7/8 of these mutations were in a poly-nucleotide regions, further suggesting sequencing error. The remaining mutation (G>T, position 1949472) was synonymous. While a hybrid assembly approach would definitively show if these mutations were due to erroneous sequencing, these data, in conjunction with our LC-MS data, leave us confident that the bactericidal phenotype exhibited by the phzA-GS*M strain is due to PYR production. We will deposit these sequences on the NCBI Sequence Read Archive in conjunction with our Pa sequences (Bioproject PRJNA1311040). Please note that we have lifted the embargo on this Bioproject to allow for review. The mutation data are summarized below:*

***Table R1. Summary of mutations identified in the phzA-GS*M and phzA-GSM strains.***

*Summary of changes: Addition of text to material and methods.*

7.      Figure 7 is missing a critical control for all panels. Please include the log fold change of each species after culture in MG1655 spent medium as well as PA14 spent medium to help the reader compare sensitivity to phenazines overproduction. Alternatively, please verify sensitivity to purchased/purified phenazines. Similarly, in Figure S14A, it would be helpful to include inhibition by PA14 spent media on this graph to support the statement in the text that "JV69 was inhibitory to a comparative level as PA14" especially as there appears to be little either limited growth of Kp in this experiment or limited inhibition by JV69.

*We have repeated these experiments with these controls (spent MG1655 media and PA14 spent media, Figure 7) and have added text reporting these results, which are consistent with our previous conclusions. Additionally, we have added the PA14 spent media data to Figure S15A. Additional text describing these results is below:*

*To assess the species-specificity of phenazine-dependent growth restriction, we repeated our heterologous expression spent media growth assays with a panel of different bacteria, specifically those that are important causes of UTI. We included two K. oxytoca strains (Ko, JV282, JV453) to test within the Klebsiella genus, two Ec strains (UTI89, CFT073) to test outside the Klebsiella genus, and an Enterococcus faecalis strain (JV25) isolated from a mouse gut to test a Gram-positive species. The effects of PCA, PYO, and PYR on Gram-negative bacteria were similar to that of Kp, wherein they exhibited growth restriction by Pa PA14 and high sensitivity to PYR and PYO, with overall sensitivity varying by strain (Figure 7C-D). The Ec strains and the Ko strain JV282 also exhibited sensitivity to PCA. Interestingly, our representative Gram-positive species, E. faecalis, did not exhibit a high degree of sensitivity to Pa PA14, PYR, or PYO, but modest sensitivity to PCA (Figure 7E). Collectively, this suggests that phenazines have greater effects on Gram-negative bacteria than Gram-positive bacteria; however, a more extensive study is required to test this premise. Nonetheless, these data indicate the effects of phenazines are not specific to Kp and the data shown here are likely applicable to a wide array of bacteria.*

*Summary of changes: Addition of controls to Figures 7 and S15A, as well as text modification explaining these results.*

8.      The observation that gut colonization by Pa 191.1 was unable to restrict colonization by KPPR1 is important given the robust restriction *in vitro*. The authors note that this could be due to poor availability of environmental oxygen, and describe the change in mouse vendor in the discussion. However, the results section seems to abruptly shift to testing a potential role for the intact gut microbiota under anaerobic conditions and then interactions in other body sites without further examining the discrepancy. It would have been interesting to see the outcome of the *ex vivo* large intestinal content experiment if conducted under aerobic conditions instead of anaerobic, or determining whether purified PYO or PYR could inhibit KPPR1 under *ex vivo* conditions. Either one of these experiments would be an important addition to the body of work to attempt to further reconcile differences between the *in vivo* and *ex vivo* studies with the *in vitro* studies and proposed role of oxygen versus other potential confounding factors. It would also be useful to note whether the Pa isolates produced phenazines under the *ex vivo* culture conditions, as lack of production could also explain the lack of Kp growth restriction.

*The purpose of our original mouse study where 145.1, 191.1, and 193.1 were isolated was to identify Kp gut fitness factors that are dependent on the presence or absence of the unperturbed gut microbiota. Thus, our aim with this ex vivo experiment was to closely replicate these conditions (intact gut microbiome, which is characteristically anoxic), which we hypothesized may be necessary to replicate our phenotype. Based on our data, we do not find evidence that this interaction occurred in the gut under our in vivo/ex vivo conditions; we now interpret the original observation as likely context-dependent and not reproducible in the gut environment. Rather, it is likely that another group of microbes are responsible for the loss of Kp colonization, and indeed, this is a major focus of our lab.*

*Nonetheless, we repeated our ex vivo conditions with pure PYO both aerobically and anaerobically (****Figure R2****). We chose 250 μg/mL, which is at or above the MIC in both aerobic and anerobic conditions, but below bactericidal concentrations (see* ***Figure 4A-D****), to ensure we would observe an effect. We spiked PYO into ex vivo large intestinal contents (with the microbiota intact) and then inoculated these samples with Kp. As expected, PYO was highly restrictive of Kp growth at this concentration under aerobic conditions. We did see a mild effect of PYO at the high concentration (10x what PA14 secretes in our experiments) under anaerobic conditions; however, there is a technical hurdle with these experiments. Kp does not grow as robustly in ex vivo large intestinal contents under anaerobic conditions due to the lack of readily available electron acceptors, so it is challenging to compare the magnitude of these differences. Given this limitation, we have decided to include these data as a reviewer-only figure, as it would require much more extensive interrogation to normalize Kp growth rates in these conditions, which is beyond the scope of this study, to confidently compare the magnitude of PYO activity. As such, and in response to point 7 from reviewer 2, we have also tested PYO activity in media under anaerobic conditions with an alternative electron acceptor (NaNO_3_) present. The goal of this experiment was to determine if the addition of an alternative electron acceptor would rescue PYO activity in anaerobic conditions. It did not, and these data are present in Figure S12C. Given that we observe that phenazine-dependent restriction requires oxygen across our media conditions in experiments that are suggested and described in reviewer comments above, we are more confident in the conclusion that our phenotypes are dependent on environmental oxygen, rather than other confounding factors. We have added additional text to the manuscript describing these results, which is below:*

*Some studies have shown that PYO remains active under anoxic conditions [55-57]. These findings suggest that PYO can remain chemically redox-active in the absence of oxygen; however, antimicrobial effects may still be oxygen-linked if they require oxygen-mediated redox cycling/ROS generation or maintenance of PYO in its oxidized state. To determine if this is the case for KPPR1, we measured the MIC of PCA, 1-HP, and PYO under anaerobic conditions. We observed a significant reduction of PYO activity under anaerobiosis in both fresh (Figure 4C) and spent (Figure 4D) media. Additionally, we repeated our co-culture competition assays under anaerobic conditions. As with our MIC studies, we did not observe Pa growth restriction of KPPR1 (Figure S12A). We also repeated our spent media growth assays using the heterologous phenazine expression system. Like our co-culture results, we did not observe any growth restriction or bactericidal activity in the case of PYR under anaerobic conditions (Figure S12B).*

*Finally, to determine if phenazine activity is dependent on respiration, we tested the MIC of PYO in conditions that favor anaerobic respiration. To this end, we repeated our MIC experiments under anaerobic conditions but grew Kp in M9 minimal medium with 0.5% glucose (“M9-glu”), conditions in which Kp predominantly undergoes fermentation, and M9-glu supplemented with 10 mM NaNO_3_, which permits anaerobic respiration by nitrate acting as an alternative electron acceptor [58, 59]. Addition of nitrate had no effect on the PYO MIC (Figure S12C), indicating that nitrate-permitting anaerobic respiration does not restore PYO activity. Thus, in our system, PYO antimicrobial action may require oxygen-dependent redox cycling or maintenance of oxidized PYO, supporting dependency on environmental oxygen.*

***Figure R2. PYO is highly restrictive of Kp growth in ex vivo large intestinal contents in aerobic conditions, but only mildly restrictive in anaerobic conditions.*** *Large intestinal contents from C57Bl6/J (no antibiotic treatment, N = 5) mice were collected and resuspended in sterile PBS. 1x10^6^ CFU KPPR1 was inoculated into large intestinal contents with or without 250 μg/mL PYO and grown aerobically or anaerobically. KPPR1 density was measured at 24 hours.*

*Summary of changes: Addition of MIC data to Figure S12 and corresponding text.*

9.      While examining competition between isolates from different body sites adds breadth to this body of work, it also leaves the reader wanting to know more about potential strain differences. For example, since the Pa strains isolated from blood and urine were more restrictive to Kp growth, it would be useful to know whether these strains tended to produce higher levels of phenazines than those from other sources, or whether their genomes cluster more closely with highly restrictive isolates like PA14 than other Pa isolates.

*We completely agree that this is an interesting and likely fruitful idea. Unfortunately, we do not have the funding to complete a cohort-wide comparative genomics study, nor develop a strain library with a more comprehensive set of metadata to more confidently assess other variables (patient, environmental, etc.), as we have previously done with Klebsiella (PMID: 35915063); however, this is essentially an aim of a recent application for NIH funding. Ideally, we will have more results to report here in the coming years!*

Additional points:

1.      More information is needed regarding the study from Supplemental 1A that provided the foundation for this body of work. For example, how/why was it hypothesized that it was a gut bacterium causing the decrease in Kp colonization, what was the rationale for selecting the three noted mice, and what lead to selection of the isolates that ultimately turned out to be Pa?

*We have revised this section to provide more detail. The revised text is below:*

*In the process of performing in vivo experiments unrelated to this study, we observed the presence of an indigenous gut bacterium associated with a reduction in Kp colonization from 24 to 48 hours after oral inoculation (Figure S1A). The goal of this experiment was to assess colonization across multiple mouse vendors and barriers (specific room in which the animals are housed), both of which are known to impact the gut microbial community structure [42]. Kp colonization was tested in three vendor backgrounds (The Jackson Labs, Taconic Farms, and Charles River Labs), from mice sourced from 13 barriers, with biological sex evenly distributed. For these experiments, we used an aggressive antibiotic selection strategy for our Kp strain, which is resistant to kanamycin and rifampicin. The unidentified gut bacterium was the only non-Kp bacteria that grew on selective media that we observed in any experiment and was only observed in a single vendor facility and two barriers (Taconic Farms, Germantown facility, barriers GT-20 and GT-1503, only Taconic Farms mice are shown in Figure S1A). This bacterium was only observed in three samples, all from male mice, and exhibited identical colony morphology. We selected three representative single colonies from these strains of this bacterium at random, which we named 145.1, 191.1, and 193.1 based on the mouse of origin, and identified them as Pa based on colony morphology and matrix-assisted laser desorption ionization–time of flight (MALDI-TOF) mass spectrometry.*

*Summary of changes: Revision of text.*

2.      For the genome sequencing in Fig S1B, the text of the legend and manuscript state that these strains "were most similar to sequence type ST175" but also state that MSLT was performed. Were the strains ST175, or a different ST?

*We have revised this section to provide more detail. The revised text is below:*

*Whole-genome sequencing of these strains revealed that strain 145.1 was ST175 whereas 191.1 and 193.1 were unknown MLSTs, but matched 6/7 and 5/7 alleles to ST175 (as well as ST159, ST171, ST2798, ST2868, ST3748), respectively. These strains clustered with other ST175 Pa isolates, which grouped with Clade A strains (PAO1 major group, Figure S1B) from a previous study [43].*

*Summary of changes: Revision of text.*

3.      In the results section prior to discussing Fig 3, the authors state "We observed that several mutants in our screen appeared to have modified phenazine production. The psqA, rhlR, and tipA mutants did not produce high amounts of pyocyanin (PYO) and pyorubin (PYR)." Please provide the data supporting these statements.

*These data have been added to Figure S7, and we have added text describing these results. The revised text is below:*

*We observed that several mutants in our screen appeared to have modified phenazine production. Production of blue PYO and red PYR leads to coloration of spent culture media when in high enough concentrations. The psqA, rhlR, and tpiA mutants did not produce high amounts of pyocyanin (PYO, Figure S7F). As PYO has reported antimicrobial activity [46], we aimed to test their role in Kp restriction.*

*Summary of changes: Addition of absorbance data to Figure S7 and addition of corresponding text.*

4.      It is interesting that co-culture of Kp with Pa phzH restricts growth, but the supernatant of phzH does not restrict growth (Fig 3D vs 3E). Further discussion on possible mechanism of contact-dependent restriction by this mutant is warranted.

*We agree that the contact dependence of this restriction is interesting, especially as a similarly stringent contact dependence is not observed for other mutants. We believe this effect may be caused by overproduction of PCA. Previous studies have shown that ΔphzH mutants of Pa produce much higher concentrations of PCA than wild type cells (PMID: 26813638). Other studies have also demonstrated that the toxicity of PCA is dependent on its reduction rate (PMID: 35138927) and that the electron transport chain of Pa is capable of reducing PCA (PMID: 36840394). This suggests a contact-dependent mechanism of Kp growth restriction whereby PCA toxicity is dependent on both the overproduction and the reduction of PCA by PA14ΔphzH. However, this hypothesis is speculative and is unlikely to be impactful in our system given the low production of PCA by WT PA14. As a result, we have included the following statement in the main text:*

*…the ΔphzH mutant displayed an entirely contact-dependent growth restriction of Kp which was not observed in other mutants, suggesting PYO and PYR were not responsible for the activity of this mutant.*

*Summary of changes: Addition of text.*

5.      Please add PYR production to the diagram in Figure 3C to help further orient the reader as to why disrupting either phzA-G or phzHMS also disrupts PYR production. It is also recommend that the labels used in figure 5B (having the respective overproduced phenazine noted for each spent medium) be applied to all other relevant figure panels to help orient the reader.

*This has been added to Figure 3C, and labels have been added to the heterologous expression strains throughout the relevant figures (3F, 4F, 5B, 7A-E, S9, S11A, S11D, S12B)*

*Summary of changes: Modification of Figure 3C and addition of labels to select figures.*

6.      For the experiments described in Figure 4, the rationale for using the PA14pPhzA-G spent medium is unclear, considering that this medium should have PCA similar to the MG1655pPhzA-G culture supernatant. Doe the data then suggest that PYO synergizes with PCA or other components of the spent media? It is also unclear if these studies were conducted using purchased/synthesized PYO, or PYO from the MG1655 phzS mutant.

*We apologize for the lack of clarity. We use PA14ΔphzA-G spent LB to mimic the nutrient conditions in which Kp is experiencing phenazine-induced stress, as nutrient availability will modulate the sensitivity of Kp to phenazine by changing the cellular redox state. The PA14ΔphzA-G spent LB does not contain PCA, so it is not comparable to the* *MG1655pPhzA-G spent media. The reviewer refers to the use of “PA14pPhzA-G spent medium,” but we do not have a PA14pPhzA-G strain. Thus, these experiments do not suggest synergy with PCA or other media components but rather reflect that nutrient availability impacts the sensitivity of Kp to phenazines. We have clarified this in the text, as well as clarified when we are using synthesized versus heterologously expressed phenazines. The revised text is below:*

*To replicate our co-culture and spent media culture assays, we measured the MIC and MBC of PYO. We performed these assays in both fresh LB and PA14ΔphzA-G spent LB, the latter of which we used to mimic the nutrient conditions in which Kp is experiencing phenazine-induced stress, as nutrient availability will modulate phenazine sensitivity by changing the cellular redox state [54].*

*Summary of changes: Revision of text.*

7.      In Figure S10, not all of the strain symbols indicated in the legend can be visually detected in panels B-F.

*The reviewer is correct. We only included the knockout and heterologous expression strains in the experiments in panels B and D, respectively, to provide a reference for the amount of PYO and PYR the clinical strains are producing. We have modified the figure legend to clarify that these symbols are unique to their respective panels and moved all control symbols to the forwardmost plane to make them easier to find.*

*Summary of changes: Modification of Figure S11.*

8.      In Figure S12, it would be helpful to show the "strain alone" growth data for all Kp strains in comparison to PA14 restriction.

*These data are now available in S13A (formerly S12A).*

*Summary of changes: Modification of Figure S13A.*

9.      In Figure S13A, please indicate what the red and orange horizontal lines indicate.

*We apologize for this oversight. We have added that information to the legend text:*

*…data point is a biological replicate, and horizontal lines indicate the mean of each dataset. The red line represents the mean normalized fluorescence for KPPR1, and the orange line represents the mean normalized fluorescence for KPPR1 + PA14.*

*Summary of changes: Revision of text.*

10.     In Figure 6, were mice and organ homogenates also inoculated with KPPR1 alone? If so, the addition of these data to panels B and C would be beneficial.

*We did not include KPPR1 alone controls, as this is a published approach for generating KPPR1 growth media (PMID: 31449551).*

11.     Pa CFUs are not reported in any of the figures, with the exception of one supplemental figure. Do any of the co-culture conditions that lack Kp growth restriction exhibit differences in Pa growth?

*We have added these data in Figure 1G and added additional text describing these findings. We did not observe any significant change in Pa growth when Kp was present in co-culture. The revised text is below:*

*Co-culture with KPPR1 had no significant effect on Pa growth (Figure 1G).*

*Summary of changes: Addition of Figure 1G and corresponding text.*

12.     Please ensure that bacterial names are italicized, including when abbreviated (for example, the first paragraph of the Discussion has several references to Ec and Pa that are not italicized).

*All bacterial name abbreviations are now italicized.*

*Summary of changes: Revision of text.*

**Reviewer #2:**

This is a rigorous and very interesting study that addresses the role of phenazines in interbacterial competition. The study starts from the observation that several mice colonised with Pa show low colonisation with Kp. Restriction of Kp by Pa is investigated in depth and is shown to depend primarily on production of phenazines by Pa. Competitive outcomes vary depending on strain background and environmental conditions, and the authors present ex-vivo data suggesting that Pa-Kp antagonism is likely to vary by host niche. Overall I found the study to be thorough and well-presented and believe it will be of broad interest. The study also fills an important gap in the field by exploring interbacterial competition in mechanistic depth - although the picture it presents is very complicated, this type of study is very much needed and will eventually lead to the ability to predict competitive outcomes. I also commend the authors for their full description and thoughtful treatment of some of the unexpected results in this study (ie. that phenazine-based restriction of Kp by Pa is unlikely to occur in the mouse gut although this was suggested by the first experiment of the study). I have only minor suggestions to improve the manuscript.

*We thank the reviewer for their positive feedback and have outlined our changes and responses to suggested improvements below.*

1.      In the discussion I find the idea that this study defines "interaction rules" for phenazine-mediated competition too expansive for the data presented. The authors could broaden the experimental investigation into the effect of environment on phenazine-mediated competition *in vitro* (by examining how competition changes with added antioxidants, graded oxygen content in addition to oxic/anoxic, and presence of electron acceptors). If this is not feasible then I suggest this statement should be softened.

*The reviewer’s point that much more experimental support is required for this statement is well-taken. Our aim was to contextualize the application of these findings in predicting bacterial interaction outcomes. We have revised the text to the below:*

*As such, it is important to measure the effects of these variables when evaluating the efficacy of interventions that leverage microbial competition, when possible. Resulting principles may enable prediction of pathogen dynamics across host sites and inform development of ecological therapeutics that exploit interbacterial antagonism. If successful, such interventions have the potential to both lower the burden of infection complicated by antibiotic resistance and re-potentiate existing antibiotic therapies by reducing their use.*

*Summary of changes: Revision of text.*

2.      Introduction - The part of the paragraph starting "Redox-active natural products mediate…" should include some references.

*Citations have been added, and the text for this section has been expanded to add clarity. The new text is below:*

*Redox-active natural products mediate microbial competition across environments, and these interactions can be consequential for environmental outcomes that are dependent on the fitness of specific microbes, such as polymicrobial community structure, bacterial virulence, and infection [19-22]. One of the most important classes of redox-active natural products are the phenazines [23]. Phenazines produced by Pa, including pyocyanin, phenazine-1-carboxylic acid, and pyorubin, modulate respiration, iron acquisition, and biofilms, and display broad antimicrobial activities across kingdoms. Yet how environmental context gates their efficacy in mediating microbial competition remains unclear.*

*Summary of changes: Revision of text.*

3.      Text presentation of Figure 1D - This is confusing to present ratios on ratios, it could be simpler to compare all of the growth rates to those in fresh LB?

*We apologize for the confusion. Given that reviewer 3 felt that reporting these exact percentages could lead to overinterpretation of the data, we have omitted these exact calculations.*

*Summary of changes: Omission of text.*

4.      Section on nutrient depletion - suggest making it explicit that these are single-strain growth experiments.

*Additional text has been added to clarify our approach. We have revised the text to the below:*

*Despite having distinct carbon utilization profiles, we did not exclude the possibility that Pa is exhausting specific nutrients from both LB broth and M9-cas. Thus, we reasoned that nutrient supplementation could fully restore KPPR1 growth. We tested the ability of KPPR1 to grow in spent LB broth supplemented with casamino acids using our single-strain spent media growth assay.*

*Summary of changes: Revision of text.*

5.      The section on phenazines and their contribution to restriction of Kp is difficult to follow at times. I suggest adding a small panel to Figure 3 to state which molecules are produced by which mutant or overexpression strain (although readers can piece this together from the overview of the biosynthesis), and stating which phenazines are not responsible for restriction when relevant (eg. when discussing lack of effect of the ∆phzH mutant).

*A panel with this information has been added to Figure 3C and throughout other figures, as recommended by reviewer 1.*

*Summary of changes: Modification of Figure 3C and addition of labels to select figures.*

6.      Fix grammar in the sentence "Consistent with previous reports, the addition of reducing agent (generating reduced 5MPCA) is more active against Kp."

*We apologize for the confusion. This sentence has been revised to:*

*Consistent with previous reports, the addition of a reducing agent (generating reduced 5MPCA) increased anti-Kp activity (Figure S9).*

*Summary of changes: Revision of text.*

7.      Section on oxygen dependence of Pa restriction of Kp: it would be very interesting to know whether Kp is undergoing anaerobic respiration or fermentation in these experiments - could the authors compare restriction with and without alternative electron acceptors? Alternatively, could they comment on which strategy is more likely based on the experimental conditions?

*This is an excellent question that we are actively trying to answer. Because K. pneumoniae is a facultative anaerobe capable of both fermentation and anaerobic respiration, the metabolic mode in our assays will depend strongly on the availability of terminal electron acceptors. In preliminary work (in a separate study), we found that Kp favors anaerobic respiration when a suitable electron acceptor is available, which we observed in vivo during gut colonization in settings that are replicated in this study, and growth increases with electron-acceptor availability; however, we have not yet defined which acceptors dominate in our gut-relevant conditions. Across our media conditions, we observe that phenazine-dependent restriction requires molecular oxygen, suggesting an oxygen-dependent step in phenazine activity in our system (e.g., redox cycling/ROS generation). To confirm this in the context of a known alternative electron acceptor, we repeated our MIC studies comparing the PYO MIC in M9-glucose medium and M9-glucose supplemented with NaNO_3_. We did not observe that the addition of NaNO_3_ lowered the PYO MIC. These data are present in Figure S12, and we have added text to the manuscript describing these results. Also, as pointed out by reviewer 3, some phenazine activity may be independent of redox activity, wherein it is conditionally dependent on expression of target enzymes. Phenazine target identification remains fully unexplored in Kp but is the subject of an aim of the recent application for NIH funding referenced in response to Reviewer 1 point 9. The additional text is below:*

*Some studies have shown that PYO remains active under anoxic conditions [55-57]. These findings suggest that PYO can remain chemically redox-active in the absence of oxygen; however, antimicrobial effects may still be oxygen-linked if they require oxygen-mediated redox cycling/ROS generation or maintenance of PYO in its oxidized state. To determine if this is the case for KPPR1, we measured the MIC of PCA, 1-HP, and PYO under anaerobic conditions. We observed a significant reduction of PYO activity under anaerobiosis in both fresh (Figure 4C) and spent (Figure 4D) media. Additionally, we repeated our co-culture competition assays under anaerobic conditions. As with our MIC studies, we did not observe Pa growth restriction of KPPR1 (Figure S12A). We also repeated our spent media growth assays using the heterologous phenazine expression system. Like our co-culture results, we did not observe any growth restriction or bactericidal activity in the case of PYR under anaerobic conditions (Figure S12B).*

*Finally, to determine if phenazine activity is dependent on respiration, we tested the MIC of PYO in conditions that favor anaerobic respiration. To this end, we repeated our MIC experiments under anaerobic conditions but grew Kp in M9 minimal medium with 0.5% glucose (“M9-glu”), conditions in which Kp predominantly undergoes fermentation, and M9-glu supplemented with 10 mM NaNO_3_, which permits anaerobic respiration by nitrate acting as an alternative electron acceptor [58, 59]. Addition of nitrate had no effect on the PYO MIC (Figure S12C), indicating that nitrate-permitting anaerobic respiration does not restore PYO activity. Thus, in our system, PYO antimicrobial action may require oxygen-dependent redox cycling or maintenance of oxidized PYO, supporting dependency on environmental oxygen.*

*Summary of changes: Addition of data to Figure S12 and corresponding text.*

8.      The reconciliation of the initial gut colonisation observations with further experiments is particularly thoughtful and thorough.

*We are pleased that the reviewer feels that way!*

**Reviewer #3 (Wulf Blankenfeldt):**

The manuscript Environmental redox conditions tune phenazine-mediated bacterial antagonism by Todd, Schneider and colleagues deciphers the antagonistic activity of clinical isolates of *Pseudomonas aeruginosa* against *Klebsiella pneumoniae*. The work described in this manuscript is based on a different study from the authors' laboratory in which they have observed this antagonism caused by a kanamycin and rifampicin resistant bacterium, then identified as three different strains of *P. aeruginosa*.

The authors then follow with a convincing cascade of experiments, showing that the antagonism is triggered by phenazine derivatives synthesized by *P. aeruginosa* (namely pyocyanin and the lesser-known pyorubin(s)). They also show that this is not a "black-and-white" finding, but that the ability of *P. aeruginosa* to kill/stall *K. pneumoniae* depends on the environmental conditions, probably requiring the availability of oxygen and the absence of substances that can alleviate oxidative stress. Further, they demonstrate that not all *P. aeruginosa* strains can antagonize *Klebsiella* to the same extent. They also provide evidence that gram-negatives seem to be generally more sensitive to the *P. aeruginosa* phenazines than gram-positive bacteria.

The manuscript is well written and I enjoyed following the authors' story. I have only very minor remarks to make since I cannot really assess the quality of the experimental data because I work in a different research field:

*We thank the reviewer for their kind words and have outlined our changes and responses to suggested improvements below.*

-       In the sentence "The growth of Kp strain KPPR1 was restricted 99.4%, 98.7%, and 97.5% by Pa 145.1, 191.1, and 193.1, respectively (Figure 1A)." on page 15 of the review document, I was wondering if the percentage numbers are stated a little too precisely and if they should not have standard deviations as well.

*Reviewer 2 felt that reporting these exact percentages were confusing to interpret (see reviewer 2, comment #3); thus, we have omitted these exact calculations.*

*Summary of changes: Omission of text.*

-       On page 22 (analysis of transposon mutants), I was surprised that the authors have not also identified PqsE as one of the required factors, since it is quite well established that PqsE (the PQS response protein) is important/required for phenazine biosynthesis in *P. aeruginosa*. On the same page, my feeling was that the authors simplify the situation around phenazine biosynthesis in *P. aeruginosa* somewhat: *P. aeruginosa* has two phz-operons that will produce phenazine-1-carboxylic acid, the substrate for pyocyanin- and pyorubin-producing enzymes. There is no specification if they found transposon mutations in both operons.

*We were also surprised by the finding that more known phenazine biosynthesis genes and regulators were absent in our screen; however, there are some potential explanations. First, regarding PqsE, this mutant did not meet our significance criteria, but did trend towards a less inhibitory phenotype. Our assumption is if we repeated our screen to reduce trial-to-trial variability, this would result in this gene being a candidate for validation. Second, regarding the phz loci, there are many phz mutants in the library; however, mutations in these genes were insufficient to disrupt inhibitory activity. We assume that there is compensatory regulation of these loci when one is interrupted, and indeed the phzA1 transposon mutant was a candidate mutant that inhibited Kp more than the mean, suggesting interruption of the phzA-G1 locus leads to overexpression of phzA-G2. Interestingly, mutation of phzA2 did not yield reciprocal results. Nonetheless, these findings are supported by the loss of Kp growth inhibition when both loci are deleted, as with our PA14ΔphzA-G mutant. We have added text to the manuscript to describe these findings in more detail and expanded the explanation of phenazine biosynthesis in this context. The revised the text to the below:*

*Then, we repeated our co-culture and spent media assays with strains containing deletions in the phzA-G1-2, phzH, phzM, phzS, and phzHMS loci. PhzA-G are necessary to synthesis phenazine-1-carboxylic acid (PCA), which is converted to 5MPCA by PhzM and from 5MPCA to PYO by PhzS or directly converted from PCA to 1-hydroxyphenazine (1-HP) by PhzS [49]. Additionally, PhzH converts PCA to phenazine-1-carboxamide (PCN). Pa encodes two phzA-G loci, and deletion of both loci renders Pa unable to synthesize PCA, and thus PYO, 5MPCA (and thus, PYR), 1-HP, or PCN. Deletion of phzH ablates PCN production, deletion of phzS ablates PYO and 1-HP production, deletion of phzM ablates 5MPCA and PYO, and deletion of phzHMS ablates production of PYO, PYR, 1-HP, and PCN but maintains PCA production (Figure 3C). Interestingly, we observed significantly less Kp growth restriction by the ΔphzA-G, ΔphzM, ΔphzS, and ΔphzHMS mutants in co-culture, and no impact with the ΔphzH mutant (Figure 3D). Similar results were observed in our spent media assay, though we did observe significantly less growth restriction in ΔphzH-spent LB broth (Figure 3E). Of note, we did not identify any phz mutant in our initial transposon screen. Given that one phzA-G locus is sufficient for phenazine production, we would not expect to identify these mutants as candidates. In fact, phzA1 (PA14_09480) was a candidate that inhibited Kp more than average, suggesting that there may be overexpression of phenazines; however, this observation failed to validate following the screen (Figure S7D-E). Additionally, the known phenazine biosynthesis regulator PqsE did not meet our significance criteria, but the data fit the general trend of a less inhibitory strain (trial 1 z-score = 2.13, trial 2 z-score = 1.19). We were surprised that the phzM or phzS mutants were not identified our transposon screen, as these mutants should have disrupted phenazine biosynthesis; however, these transposon interruptions may not sufficiently inactive gene function, whereas clean knockouts yielded higher-confidence data (Figure 3D). Collectively, this suggests that the phenazine PYO and/or PYR are necessary for the observed growth restriction phenotype.*

*Summary of changes: Addition of text.*

-       On page 23, the authors state that "Due to the chemical similarity between PYO and other phenazines, it is commonly assumed that these compounds exert similar biological effects." This may be correct, but it is quite well established in the literature that different phenazines have different redox potential. In addition, there are now several examples of phenazines not acting via their redox activity but through other modes of action (for example this one: <https://nam12.safelinks.protection.outlook.com/?url=https%3A%2F%2Fpubmed.ncbi.nlm.nih.gov%2F40935925%2F&data=05%7C02%7Cjayvornh%40iu.edu%7Ca958d7139ee54b177e1708de4d3f4206%7C1113be34aed14d00ab4bcdd02510be91%7C1%7C0%7C639033131294613591%7CUnknown%7CTWFpbGZsb3d8eyJFbXB0eU1hcGkiOnRydWUsIlYiOiIwLjAuMDAwMCIsIlAiOiJXaW4zMiIsIkFOIjoiTWFpbCIsIldUIjoyfQ%3D%3D%7C0%7C%7C%7C&sdata=TxVuZ56Gi6BqBOjQX994pT9mZ8vKs%2FltLjVqGWhpwxQ%3D&reserved=0)>

*Upon reviewing this statement, we have decided it is overly speculative and thus, have omitted it.*

*Summary of changes: Omission of text.*

-       On page 27, the authors report the amount of pyocyanin that different strains *of P. aeruginosa* produce. I wonder if these numbers can be correct. For example, if there were indeed 20.7 mg/ml from P. aeruginosa PA14, this would amount to 20.7 g per liter of culture, roughly 0.1 Mol. This sounds like an awful lot, but I may be mistaken. However, such high concentrations would indicate that pyocyanin/pyorubin are very ineffective bacteriostatics/antibiotics. In this context, the authors may want to read a little on "pyocyanase", an antibiotically active concoction from *P. aeruginosa* marketed in the pre-antibiotic era.

*This is a typo. The unit is μg/mL, not mg/mL. This has been corrected.*

*Summary of changes: Correction of text.*

-       Still at this same spot on page 27, I would also appreciate if the authors could state the concentrations also in molar units.

*Molar concentrations have been added to the text. The revised text reads:*

*PYO results were concordant with our co-culture and spent media assays, wherein PAO1 produced the least PYO (0.272 μg/mL [1.29 μM]) and was the least restrictive strain, followed by 145.1 (4.02 μg/mL [19.1 μM]), 191.1 (15.5 μg/mL [73.7 μM]), 193.1 (17.4 μg/mL [82.8 μM]), and PA14 (20.7 μg/mL [98.5 μM], Figure 4E).*

*Summary of changes: Addition of text.*

-       On page 39, I was not sure what the authors mean by "true, true, unrelated".

*The text has been revised to:*

*However, our attempts to recapitulate these findings suggest that our original observation was happenstance, wherein Pa could have been a contaminant or transient gut colonizer…*

*Summary of changes: Revision of text.*

**Journal Requirements:**

1) Please send a completed 'Competing Interests' statement, including any COIs declared by your co-authors. If you have no competing interests to declare, please state "The authors have declared that no competing interests exist". Otherwise please declare all competing interests beginning with the statement "I have read the journal's policy and the authors of this manuscript have the following competing interests:"

*This has been completed.*

2) Please provide separate figure files in .tif or .eps format.

*These are provided with the resubmission.*

3) We notice that your supplementary figures and tables are included in the manuscript file. Please remove them and upload them with the file type 'Supporting Information'. Please ensure that each Supporting Information file has a legend listed in the manuscript after the references list.

*This has been changed.*

4) Please refer to the following article for PLOS Biology's best-practice recommendations for data presentation and statistics.

*This have been reviewed and our manuscript is in accordance with these recommendations.*

5) Some material included in your submission may be copyrighted. According to PLOS’s copyright policy, authors who use figures or other material (e.g., graphics, clipart, maps) from another author or copyright holder must demonstrate or obtain permission to publish this material under the Creative Commons Attribution 4.0 International (CC BY 4.0) License used by PLOS journals. Please closely review the details of PLOS’s copyright requirements here: PLOS Licenses and Copyright. If you need to request permissions from a copyright holder, you may use PLOS's Copyright Content Permission form.

Please respond directly to this email and provide any known details concerning your material's license terms and permissions required for reuse, even if you have not yet obtained copyright permissions or are unsure of your material's copyright compatibility. Once you have responded and addressed all other outstanding technical requirements, you may resubmit your manuscript within Editorial Manager.

Potential Copyright Issues:

i) S7A: Please confirm whether you drew the images / clip-art within the figure panels by hand. If you did not draw the images, please provide (a) a link to the source of the images or icons and their license / terms of use; or (b) written permission from the copyright holder to publish the images or icons under our CC BY 4.0 license. Alternatively, you may replace the images with open source alternatives. See these open source resources you may use to replace images / clip-art:

*This figure was generated in BioRender, and we have added that information in the figure legend, as per BioRender citation guidelines (*[*https://help.biorender.com/hc/en-gb/articles/17605511350685-How-to-cite-your-BioRender-figure)/*](https://help.biorender.com/hc/en-gb/articles/17605511350685-How-to-cite-your-BioRender-figure)/)*.*
