# Supplementary material for: Environmental redox conditions and strain variation define phenazine-mediated antagonism in co-infecting bacteria
Source: PLoS Biol. 2026 May 20;24(5):e3003809. doi: 10.1371/journal.pbio.3003809 (PMC13197074; doi:10.1371/journal.pbio.3003809)
Supplement: Attachment — Submitted filename: Response to editor.docx [file pbio.3003809.s024.docx]

**Response to editor**

*We thank the editor for their thorough review of our manuscript. Our responses to the editor’s requests are in blue.*

**Editor requests:**

We routinely suggest changes to titles to ensure maximum accessibility for a broad, non-specialist readership, and to ensure they reflect the contents of the paper. In this case, we would suggest a minor edit to the title, as follows. Please ensure you change both the manuscript file and the online submission system, as they need to match for final acceptance:

"Environmental redox conditions and strain variation define phenazine-mediated antagonism in co-infecting bacteria"

*We have changed the title to the above.*

2) Please supply the numerical values either in the a supplementary file or as a permanent DOI’d deposition for the following figures:

Figure 1A-G, 2A-D, 3ABDEF, 4A-H, 5A-E, 6ABC, 7A-E; S1A, S2A-D, S3A-D, S4A-D, S5A-J, S6AB, S7B-F, S8A-D, S9, S11A-F, S12ABC, S13AB, S14AB, S15A, S16A-D, S17ABC

NOTE: the numerical data provided should include all replicates AND the way in which the plotted mean and errors were derived (it should not present only the mean/average values).

*All raw data for the above figures are in the supplementary information (S1-S2 Data), and we have updated our data availability statement.*

3)  Please cite the location of the data clearly in all relevant main and supplementary Figure legends, e.g. “The data underlying this Figure can be found in S1 Data.”

*The location of all raw data is now in each figure legend.*

4) For figures containing any spectrometry data (Figures 3G, S10AB, S15B), please deposit the data on publicly available databases and provide the accession number/URL of the deposition in the Data Availability Statement in the online submission form.

*All raw spectrometry data is now available on the NIH Common Fund's National Metabolomics Data Repository at* [*http://dx.doi.org/10.21228/M8D858*](http://dx.doi.org/10.21228/M8D858)*. Please note that this data has a release date of 5/21/26, but a temporary submission can be found here:* [*https://dev.metabolomicsworkbench.org:22222/data/DRCCMetadata.php?Mode=Study&StudyID=ST004803&Access=OecZ9013*](https://nam12.safelinks.protection.outlook.com/?url=https%3A%2F%2Fdev.metabolomicsworkbench.org%3A22222%2Fdata%2FDRCCMetadata.php%3FMode%3DStudy%26StudyID%3DST004803%26Access%3DOecZ9013&data=05%7C02%7Cjayvornh%40iu.edu%7C4992733cd84c41389dcb08dea6f015fb%7C1113be34aed14d00ab4bcdd02510be91%7C1%7C0%7C639131747279177742%7CUnknown%7CTWFpbGZsb3d8eyJFbXB0eU1hcGkiOnRydWUsIlYiOiIwLjAuMDAwMCIsIlAiOiJXaW4zMiIsIkFOIjoiTWFpbCIsIldUIjoyfQ%3D%3D%7C0%7C%7C%7C&sdata=Id5RvIqjL61B5bVNTn4DCbLAdOZH5hXbZwblHa%2BiPY0%3D&reserved=0)*.* *We have updated our data availability statement to reflect this change.*

5) Please provide the tree files for the phylogenetic trees in Figures S1B. Please make sure all relevant figures have scale bars.

*The tree data is available in the supplementary information (S4 Data).*

6) Supplementary files (e.g., excel). Please ensure that all data files are uploaded as 'Supporting Information' and are invariably referred to (in the manuscript, figure legends, and the Description field when uploading your files) using the following format verbatim: S1 Data, S2 Data, etc. Multiple panels of a single or even several figures can be included as multiple sheets in one excel file that is saved using exactly the following convention: S1_Data.xlsx (using an underscore).

*Our raw data conforms with the above.*

7) Please ensure that your Data Statement in the submission system accurately describes where your data can be found and is in final format, as it will be published as written there

*This has been checked and is up to date.*

8) Thank you for providing the underlying code in GitHub. However, because Github depositions can be readily changed or deleted, please make a permanent DOI’d copy (e.g. in Zenodo) and provide this URL in the manuscript and Data Availability Statement.

*A permanent DOI’d copy of the supplementary files with raw data, as well as the R-compatible versions of the data and corresponding R scripts have been uploaded to Zenodo (*[*https://doi.org/10.5281/zenodo.19501469*](https://doi.org/10.5281/zenodo.19501469)*), and this is indicated in the Data Availability Statement.*

As you address these items, please take this last chance to review your reference list to ensure that it is complete and correct. If you have cited papers that have been retracted, please include the rationale for doing so in the manuscript text, or remove these references and replace them with relevant current references. Any changes to the reference list should be mentioned in the cover letter that accompanies your revised manuscript.

*We have confirmed that references are correct and we have not cited any retracted manuscripts.*
